# Immunogenicity of a Bivalent Adjuvanted Glycoconjugate Vaccine against *Salmonella* Typhimurium and *Salmonella* Enteritidis

**DOI:** 10.3389/fimmu.2017.00168

**Published:** 2017-02-27

**Authors:** Fabio Fiorino, Simona Rondini, Francesca Micoli, Luisa Lanzilao, Renzo Alfini, Francesca Mancini, Calman A. MacLennan, Donata Medaglini

**Affiliations:** ^1^Laboratorio di Microbiologia Molecolare e Biotecnologia (LA.M.M.B.), Dipartimento di Biotecnologie Mediche, Università di Siena, Siena, Italy; ^2^GSK Vaccines Institute for Global Health S.r.l. (formerly Novartis Vaccines Institute for Global Health S.r.l.), Siena, Italy; ^3^Jenner Institute, Nuffield Department of Medicine, University of Oxford, Oxford, UK

**Keywords:** glycoconjugate vaccines, *Salmonella* Typhimurium, *Salmonella* Enteritidis, O-antigen, immunogenicity, bivalent vaccine, adjuvants

## Abstract

*Salmonella enterica* serovars Typhimurium and Enteritidis are the predominant causes of invasive non-typhoidal *Salmonella* (iNTS) disease. Considering the co-endemicity of *S*. Typhimurium and *S*. Enteritidis, a bivalent vaccine formulation against both pathogens is necessary for protection against iNTS disease, thus investigation of glycoconjugate combination is required. In the present work, we investigated the immune responses induced by *S*. Typhimurium and *S*. Enteritidis monovalent and bivalent glycoconjugate vaccines adjuvanted with aluminum hydroxide (alum) only or in combination with cytosine-phosphorothioate-guanine oligodeoxynucleotide (CpG). Humoral and cellular, systemic and local, immune responses were characterized in two different mouse strains. All conjugate vaccines elicited high levels of serum IgG against the respective O-antigens (OAg) with bactericidal activity. The bivalent conjugate vaccine induced systemic production of antibodies against both *S*. Typhimurium and *S*. Enteritidis OAg. The presence of alum or alum + CpG adjuvants in vaccine formulations significantly increased the serum antigen-specific antibody production. The alum + CpG bivalent vaccine formulation triggered the highest systemic anti-OAg antibodies and also a significant increase of anti-OAg IgG in intestinal washes and fecal samples, with a positive correlation with serum levels. These data demonstrate the ability of monovalent and bivalent conjugate vaccines against *S*. Typhimurium and *S*. Enteritidis to induce systemic and local immune responses in different mouse strains, and highlight the suitability of a bivalent glycoconjugate formulation, especially when adjuvanted with alum + CpG, as a promising candidate vaccine against iNTS disease.

## Introduction

*Salmonella enterica* serovars Typhimurium (*S*. Typhimurium) and Enteritidis (*S*. Enteritidis) are the predominant causes of invasive non-typhoidal *Salmonella* (iNTS) disease, a bloodstream infection with high prevalence in sub-Saharan Africa, especially among young children and HIV-infected individuals (1, 2). The case fatality rate of iNTS disease is around 20%, and the effectiveness of antibiotic treatment is hampered by a growing frequency of multidrug resistance (2, 3). No vaccine against iNTS disease is currently available and, in the ongoing efforts to identify protective antigens, lipopolysaccharide (LPS) has been recognized as an important target of the protective immune response (4–6). LPS is composed of lipid A (endotoxin), attached to the 3-deoxy-D-manno-octulosonic acid (KDO) terminus of the conserved core region, which is linked to a variable O-antigen (OAg) chain containing serogroup-specific repeating units (7). The OAg of *S*. Typhimurium and *S*. Enteritidis contain a common trisaccharide backbone and a serogroup-specific side chain, providing O:4,5 and O:9 specificities for *S*. Typhimurium and *S*. Enteritidis, respectively. Although the OAg chain and core sugar alone constitute a poor immunogen, OAg conjugated to a carrier protein can elicit protective immunity against lethal challenge, with anti-OAg antibodies effective in adoptive transfer experiments (8–11). In previous work, we have shown that OAg-CRM_197_ glycoconjugates induce functional antigen-specific serum antibody in mice (6, 12) and that conjugation parameters, such as polysaccharide structural fine specificities, polysaccharide/carrier protein ratio, and conjugation chemistry, can all influence the immunogenicity of such glycoconjugates (13–15).

Considering the frequent co-endemicity of *S*. Typhimurium and *S*. Enteritidis serovars, a bivalent vaccine formulation against both pathogens would be of critical importance. Glycoconjugate combinations represent a promising approach to vaccine development. The development of immune responses by a glycoconjugate vaccine can be influenced by adjuvants that can enhance the immunogenicity of the vaccine antigens, improve vaccine efficacy in newborns, elderly, and immunocompromised individuals, and facilitate the uptake of antigens by the mucosa (16). Adjuvants can achieve qualitative modulation of the immune response and promote types of immunity not effectively generated by the non-adjuvanted antigens (17). Here, aluminum hydroxide (alum) and unmethylated cytosine-phosphorothioate-guanine oligodeoxynucleotide (CpG ODN) adjuvants were tested. Alum induces a Th2-biased immune response with predominantly B-cell-related humoral immunity (18). It has been approved by the Food and Drug Administration for formulation with human vaccines and has been in use for the past 60 years, indicating an excellent safety profile (19, 20). CpG ODN are potent stimulators of both innate and adaptive immune responses through the Toll-like receptor 9 (TLR9) (21, 22). CpG ODN stimulate cellular and humoral responses, strongly promote Th1 responses (23, 24), and have been licensed for cancer immunotherapy (25). More recently, the combination of antigens with more than one adjuvant, known as the adjuvant system approach, has been used to develop vaccines generating improved immune responses (26, 27).

In the present work, we investigated the immune responses induced by subcutaneous immunization with *S*. Typhimurium and *S*. Enteritidis glycoconjugate vaccines by characterizing humoral and cellular, systemic and local, immune responses in two different mouse strains, CB6F1 and C57BL/6. We tested monovalent (O:4,5-CRM_197_ or O:9-CRM_197_) and bivalent (O:4,5-CRM_197_ and O:9-CRM_197_) vaccine formulations, adjuvanted with alum only or in combination with CpG ODN. Antigen-specific antibodies in serum, intestinal washes and feces, serum bactericidal activity (SBA), and cytokine production in restimulated splenocytes were analyzed. The results obtained show the suitability of a bivalent adjuvanted glycoconjugate formulation as a promising candidate vaccine against iNTS disease.

## Materials and Methods

### Antigens

#### Origin of Bacterial Strains

The clinical isolate *S*. Typhimurium D23580 was obtained from the Malawi-Liverpool-Wellcome Trust Clinical Research Programme, Blantyre, Malawi. D23580 is a representative Malawian isolate belonging to ST313 sequence type isolated from a case of iNTS disease (28–30). *S*. Enteritidis CMCC4314 (corresponding to ATCC4931) was obtained from the Novartis Master Culture Collection. *S*. Typhimurium 2189 and 1418 (14) were obtained from University of Calgary, Canada (*Salmonella* Genetic Stock Centre): 2189 belonged to the *Salmonella* reference collection A (SARA) (31) and 1418 belonged to the LT2-collection (32). *S*. Enteritidis 618 (14) was obtained from Quotient Bioresearch Limited, UK and was isolated by the European Antimicrobial Susceptibility Surveillance in Animals, coordinated by the European Animal Health Study Centre, Brussels (CEESA) (33).

#### Synthesis and Characterization of Conjugate Vaccines

OAg were purified from *S*. Typhimurium 2189 and 1418 and from *S*. Enteritidis 618 strains and fully characterized as previously reported (14, 34, 35). Those strains were selected as potential sources of OAg for OAg-conjugate vaccines (14). For conjugation to CRM_197_ (received by Novartis Vaccines), OAg was derivatized with adipic acid dihydrazide (ADH) by reductive amination of the KDO terminal sugar and linked to the amino groups on the protein after attachment of a second linker, adipic acid bis(*N*-hydroxysuccinimide) (SIDEA), to ADH. Conditions used for conjugation and assays for characterization of OAg-CRM_197_ conjugates were as previously described (13–15). The main characteristics of the conjugates tested in this study are listed in Table 1. All conjugates had no free protein detectable (by HPLC-SEC) (36) and less than 20% free saccharide (following precipitation of the conjugate with deoxycholate) (37). HPLC-SEC profiles of the conjugates indicated a clear shift at higher molecular weight with respect to unconjugated protein and OAg, as shown by lower Kd (distribution coefficient) values reported in Table 1.

**Table 1:** **Characterization of O-antigen (OAg)–CRM_197_ conjugates tested in mice**.

Conjugate	OAg/CRM_197_ (w/w ratio^a^)	Free OAg (%)	Kd (SEC)
1418 O:4,5-CRM_197_	2.2	12.4	0.40
2189 O:4,5-CRM_197_	1.2	<20	0.49
618 O:9-CRM_197_	2.3	17.9	0.47
Unconjugated CRM_197_	–	–	0.72
Unconjugated 1418 O:4,5	–	–	0.56 and 0.67
Unconjugated 2189 O:4,5	–	–	0.57 and 0.68
Unconjugated 618 O:9	–	–	0.62

*^a^OAgs to CRM_197_ ratios were calculated based on OAg concentration by phenol sulfuric assay and protein concentration by micro BCA. Amount of free OAg was calculated following conjugate precipitation with deoxycholate. Kd (distribution coefficient) values were calculated by HPLC-SEC analysis on TSK gel 6000-5000 PW columns (flow rate 0.5 ml/min; eluent 100 mM NaH_2_PO_4_, 100 mM NaCl, 5% CH_3_CN pH 7.2), using the following equation: Kd = (Te − T0)/(Tt − T0), where Te = elution time of the analyte, T0 = elution time of the biggest fragment of λ-DNA, and Tt = elution time of NaN_3_*.

### Antigens Formulation

NTS glycoconjugates were diluted in saline or formulated with either alum only (Alhydrogel, Brenntag Biosector, Denmark) (2 mg/ml final concentration, measured as alum content) or with both alum (2 mg/ml) and CpG ODN 1826 (hereafter CpG; Invitrogen, 5′-TCCATGACGTTCCTGACGTT-3′; 0.1 mg/ml final concentration) in saline, as detailed in Table 2. Each formulation was stirred for 1 h at room temperature, and the tubes were mixed again before injecting into mice.

**Table 2:** **Vaccine formulations used for immunogenicity studies in CB6F1 and C57BL/6 mice**.

Mice strain^a^	Group (number of mice per group)	Vaccine formulation			
		Immunogen^b^	Adjuvant	Dose of O-antigen (μg/mouse)	Dose of CRM_197_ (μg/mouse)
CB6F1	1 (8)	O:4,5-CRM_197_	–	8	6.8
	2 (8)	O:9-CRM_197_	–	8	3.5
	3 (8)	O:4,5-CRM_197_ + O:9-CRM_197_ (bivalent)	–	8 + 8	10.3 (6.8 + 3.5)
	4 (8)	O:4,5-CRM_197_ + O:9-CRM_197_ (bivalent)	Alum	8 + 8	10.3 (6.8 + 3.5)
	5 (8)	O:4,5-CRM_197_ + O:9-CRM_197_ (bivalent)	Alum + CpG	8 + 8	10.3 (6.8 + 3.5)
	6 (6)	O:4,5	–	8	–
	7 (6)	O:9	–	8	–
	8 (6)	CRM_197_	–	–	10.3
	9 (6)	Saline	–	–	–
C57BL/6	1 (8)	O:4,5-CRM_197_	Alum	8	6.8
	2 (8)	O:4,5-CRM_197_	Alum + CpG	8	6.8
	3 (8)	O:9-CRM_197_	Alum	8	3.5
	4 (8)	O:9-CRM_197_	Alum + CpG	8	3.5
	5 (8)	O:4,5-CRM_197_ + O:9-CRM_197_ (bivalent)	Alum	8 + 8	10.3 (6.8 + 3.5)
	6 (8)	O:4,5-CRM_197_ + O:9-CRM_197_ (bivalent)	Alum + CpG	8 + 8	10.3 (6.8 + 3.5)
	7 (8)	–	Alum + CpG	–	–

*^a^CB6F1 mice were used to study long-term humoral and cellular, local and systemic, immune responses and functional activity; C57BL/6 mice were used to study humoral systemic immune responses and functional activity*.

*^b^O:4,5: O-antigen from *S*. Typhimurium (2189 for CB6F1 and 1418 for C57BL/6 mice); O:9: O-antigen from *S*. Enteritidis; CRM_197:_ non-toxic mutant of diphtheria toxin*.

*Alum, alhydrogel; CpG, cytosine-phosphorothioate-guanine oligodeoxynucleotide*.

Analysis by HPLC-SEC revealed that, at the concentrations tested in the study, less than 2% CpG was attached to alum and in both formulations much less than 10% conjugates remained free in the supernatant.

### Animal Studies

Groups of 6 week-old female CB6F1 mice and 5 week-old female C57BL/6 were purchased from Charles River Laboratories (Lecco, Italy). CB6F1 stain is a chimeric strain of mice (BALB/c  × C57BL/6), used to evaluate both humoral and cellular immune responses; C57BL/6 is a Th1-polarized inbred strain. Animals were maintained under specific pathogen-free conditions in the animal facilities at the Laboratory of Molecular Microbiology and Biotechnologies (LA.M.M.B.) at University of Siena or at Novartis Animal Facility in Siena. All animal protocols were approved by the local animal ethics committees (authorization N. 4/2011, July 20, 2011 by “Comitato Etico Locale dell’Azienda Ospedaliera Universitaria Senese” for CB6F1 mice and approval N. AEC201018 for C57BL/6 mice) and by the Italian Minister of Health in accordance with Italian law (Decreto Legislativo 26/2014).

### Immunizations and Sample Collection

**Figure 1:**
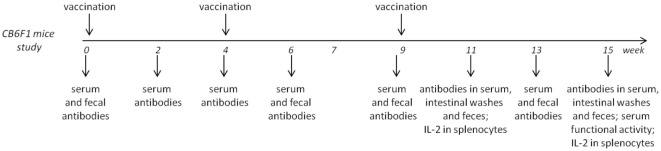
**Experimental design for immunogenicity studies with CB6F1 mice**.

Two immunogenicity studies were conducted as described in Figure 1, Figure S1A in Supplementary Material, and Table 2.

Groups of eight or six CB6F1 mice were immunized at weeks 0 and 4. Half of the animals for each group were sacrificed at week 11; the other half received a further immunization at week 9 and were sacrificed at week 15. Groups of eight C57BL/6 mice were immunized at weeks 0 and 4 and sacrificed at week 7. In both studies, each immunization was administered subcutaneously in a volume of 200 μl/mouse.

*Blood samples* were taken from the temporal plexus (submandibular vein) on weeks 0, 2, 4, 6, 9, 11, 13, and 15 (for CB6F1 study) and on weeks 0, 2, 4, 6, and 7 (for C57BL/6 study), incubated for 30 min at 37°C and centrifuged at 1,200 × *g* at 4°C for 15 min. Serum samples were stored at −80°C.

*Fecal samples* were collected from CB6F1 mice on weeks 0, 6, 9, 11, 13, and 15; feces evacuated from individual mice were weighed and carefully dissolved in 100 mg/ml of PBS–1% BSA (Sigma-Aldrich), centrifuged at 15,000 × *g* at 4°C for 10 min, and protease inhibitor cocktails (Sigma-Aldrich) were added to supernatants before storage at −80°C.

*Intestinal washes* were performed at the time points of sacrifice (weeks 11 and 15) of CB6F1 mice; the small intestine was removed and washed with 1 ml of PBS-1% BSA (BSA, Sigma-Aldrich) three times. Samples were centrifuged at 10,000 × *g* at 4°C for 10 min, and protease inhibitor cocktails (Sigma-Aldrich) were added to supernatants before storage at −80°C. Erythrocyte contamination was estimated by comparing erythrocyte number in intestinal washes with that of blood and were found to be too low to account for the observed intestinal antibody response.

*Spleens* were collected from each CB6F1 mouse at sacrifice, mashed through nylon screens (Sefar Italia, Italy), and washed in complete medium [cRPMI, RPMI 1640 (Gibco, USA) supplemented with 10% (v/v) fetal bovine serum (Gibco), 100 U/ml penicillin and 100 µg/ml streptomycin (Sigma-Aldrich)].

### Enzyme-Linked Immunosorbent Assay (ELISA)

#### Serum Antibodies

Serum anti-O:4,5 (from 2189 strain for study with CB6F1 mice and from 1418 strain for study with C57BL/6 mice), anti-O:9 (from strain 618), and anti-CRM_197_ IgG, IgG1, IgG2a, IgG2b, IgG2c, IgG3, IgA, and IgM were determined by ELISA (14). Maxisorp microtiter plates (Nunc, Denmark) were coated with O:4,5 (5 µg/ml), O:9 (15 µg/ml), or CRM_197_ (2 µg/ml) overnight at 4°C in a carbonate buffer, pH 9.4, in a volume of 100 µl/well. Coating was removed and plates were blocked with 200 µl/well of PBS–0.05% Tween 20–5% fat-free milk (AppliChem, Germany) for 1 h at room temperature. Plates were washed with PBS–0.05% Tween 20 (Sigma-Aldrich), and serum samples were added and titrated in twofold dilutions in duplicate or triplicate in PBS–0.05% Tween 20–0.1% BSA (diluent buffer) in 100 µl/well. After incubation for 2 h at room temperature, plates were washed, incubated for 1 h at room temperature with the alkaline phosphatase-conjugated goat anti-mouse IgG, IgG1, IgG2a, IgG2b, IgG2c, IgG3, IgA, and IgM (all diluted 1:1,000 and from Southern Biotechnology, USA) in 100 µl/well, and developed by adding 1 mg/ml of alkaline phosphatase substrate (Sigma-Aldrich) in 100 µl/well. The end point reading was performed using Versamax ELISA reader (Molecular Devices, Italy) or Synergy HT reader (Biotek Instruments, USA). Antibody titers were expressed as the reciprocal of the dilution of sample reporting the double OD value compared to the background.

#### Mucosal Antibodies

Anti-O:4,5 and anti-O:9 IgG and IgA in intestinal washes and in fecal samples were determined by ELISA, as previously described (38). As the concentration of IgG and IgA in intestinal washes is variable, the amount of anti-O:4,5 and anti-O:9 IgG or IgA was normalized to the total IgG or IgA concentration in each sample. Total IgG and IgA were determined on flat bottom Maxisorp microtiter plates coated with anti-mouse IgG or IgA (1 µg/ml; Southern Biotechnology), while anti-O:4,5 and anti-O:9 IgG or IgA were assayed on the same plates as described above. Samples were tested in twofold dilutions in duplicate in plates incubated overnight at 4°C. The concentration of total, anti-O:4,5, and anti-O:9 IgG or IgA was calculated against a standard curve of mouse myeloma standard IgG or IgA (Southern Biotechnology) determined on the same plate. The end point reading was performed using Versamax ELISA reader (Molecular Devices, Italy). Results were expressed as microgram of anti-O:4,5 and anti-O:9 IgG or IgA per milligram of total IgG or IgA.

### SBA assay

Equal volumes of mouse sera collected from CB6F1 mice at week 11 and from C57BL/6 mice at week 7, belonging to the same immunization group (Table 2), were pooled together for SBA experiments, as previously described (15). *S*. Typhimurium D23580 and *S*. Enteritidis CMCC4314 strains were used as target strains to perform SBA reactions as previously reported (14, 15). They were grown in Luria Bertani (LB) medium to log-phase (OD: 0.2), diluted 1:30,000 in PBS to approximately 3 × 10^3^ colony-forming units (CFU)/ml and distributed into sterile polystyrene U bottom 96-well microtiter plates (12.5 µl/well). Serum samples, serially diluted twofold or threefold (starting from 1:100 dilution), were added to each well (final volume 50 µl, ~620 CFU/ml). Sera were heat-inactivated and baby rabbit complement (Cedarlane CL3441) was used at 50% of the final volume. Each sample and control was tested in triplicate on three different days. One hundred microliters reaction mixtures from each well were spotted on LB-agar plates at time 0 (T0) to assess initial CFU and at 3 h (T180) after incubation at 37°C. LB-agar plates were incubated overnight at 37°C and the resulting CFU were counted the following day. Bactericidal activity was determined as serum dilutions necessary to obtain 50% CFU reduction at T180 compared with T0. Serum titers equal to 1 were designated when no bactericidal activity was detected.

### T ELISPOT

IL-2 and IFN-γ production was analyzed in splenocytes, as previously described (39), following two immunizations (week 11). The number of spot-forming units (SFU) was evaluated using the Mouse IL-2 and Mouse IFN-γ ELISPOT ready-SET-Go kits, according to the manufacturer’s protocol (Affymetrix eBioscience). Ninety-six-well multiscreen filtration plates (Millipore, USA) were coated overnight at 4°C with capture IL-2 or IFN-γ antibody solution. Free binding sites were blocked with cRPMI. Pooled splenocytes for each group (three to four mice per group) were seeded in triplicate in a final volume of 200 µl/well of cRPMI at a density of 1 × 10^6^ cells/well, as determined in a pilot study using different cell densities in a range suggested by the manufacturer’s protocol. Cells were stimulated with 10 µg/ml of CRM_197_ (T-dependent antigen) or of the respective OAg (T-independent antigen) and incubated for 40 h at 37°C with 5% CO_2_. A positive and a negative control [splenocytes *in vitro* restimulated with 10 µg/ml concanavalin A (Sigma-Aldrich) or unstimulated, respectively] were included for each group of immunization. After washing steps, plates were incubated with biotinylated IL-2 or IFN-γ detection antibody at room temperature for 2 h. Plates were added with avidin-HRP solution for 45 min and developed with AEC Substrate Solution (Sigma-Aldrich) at room temperature. Spots were counted using a computer-assisted ELISPOT image analyzer (Cellular Technologies Limited, Germany). Data were expressed as SFU per million of cells.

### Statistical Analysis

Sera, fecal samples, and intestinal washes for ELISA were tested individually, and the amount of antibodies were expressed as geometric mean titers (GMT) or concentration ± standard error of the mean (SEM). Sera for SBA were tested as a pool for each group of immunization and data were expressed as SBA titers ± SEM. Statistical differences between antibody production among groups were assessed using one-way analysis of variance (ANOVA) and Tukey’s post test for multiple comparisons. Two-tailed Student’s *t*-test was used for analyzing antibody amounts at two different time points or for evaluating statistical differences among two independent groups. Statistical analysis of titer values was performed on log-transformed data. The correlation among the antibody concentration between sera and fecal samples or intestinal washes in each animal was performed calculating Pearson’s correlation coefficient, *r*. Statistical significance was defined as *P* ≤ 0.05. Graphpad 4.0 software was used for analysis.

## Results

### Serum Antibody Responses in Mice Immunized with Adjuvanted and Unadjuvanted Conjugate Vaccines

Serum antibody response induced by monovalent *S*. Typhimurium (O:4,5-CRM_197_) and *S*. Enteritidis (O:9-CRM_197_) conjugate vaccines or by bivalent (O:4,5-CRM_197_ + O:9-CRM_197_) conjugate formulations, unadjuvanted or adjuvanted with alum only, or with alum plus CpG, was analyzed in CB6F1 mice immunized with the different vaccine formulations (Table 2) according to the experimental design reported in Figure 1.

**Figure 2:**
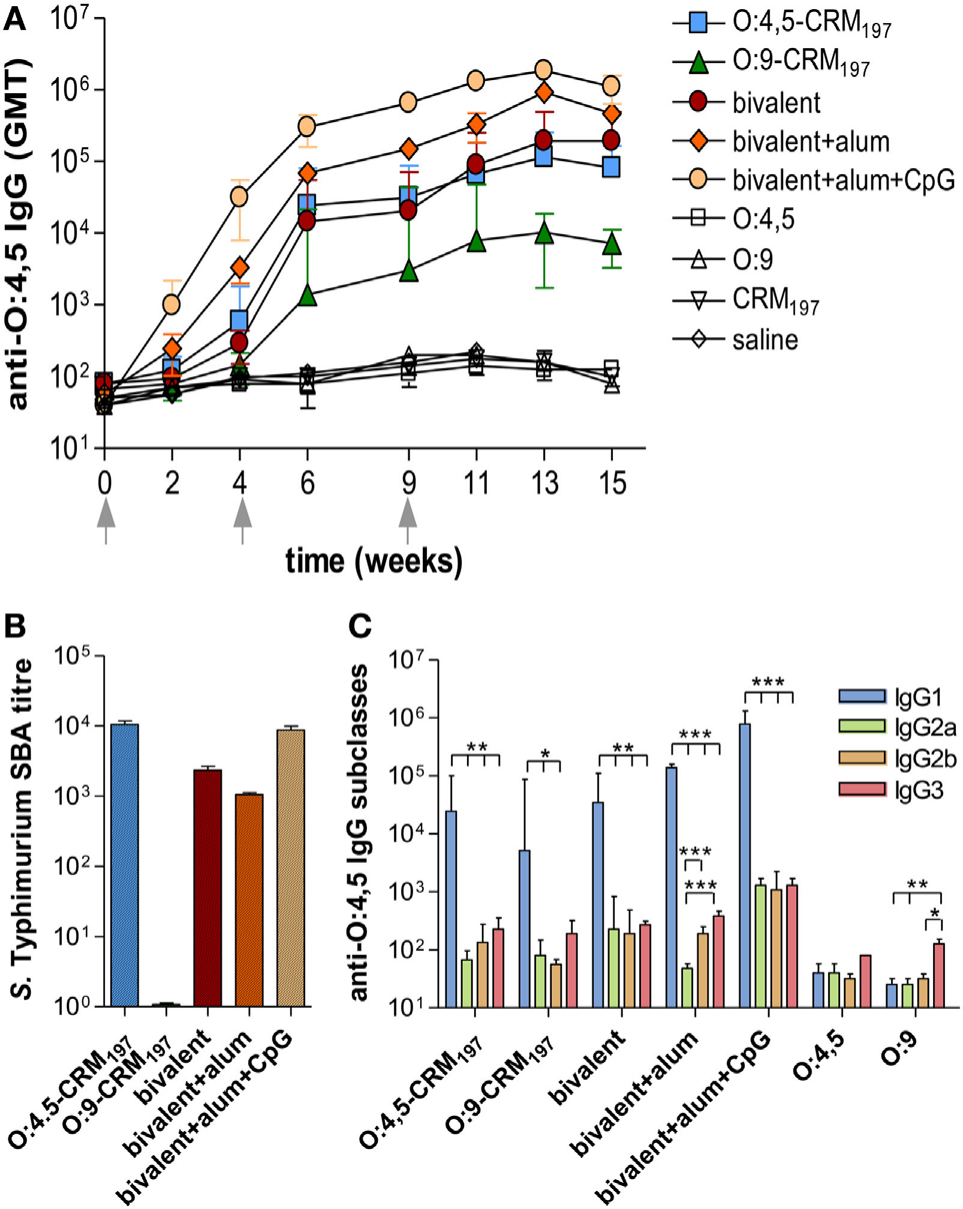
***S*. Typhimurium O-antigen-specific serum IgG, serum bactericidal activity (SBA), and IgG subclasses in CB6F1 mice**. Mice were subcutaneously immunized at weeks 0, 4, and 9 with different vaccine formulations, as reported in Table 2. **(A)** Time course of anti-O:4,5 IgG assessed by ELISA in individual serum samples on weeks 0, 2, 4, 6, 9, 11, 13, and 15 following the first immunization. Arrows represent the timing of immunizations. Values are reported as geometric mean titers (GMT) ± SEM. **(B)** SBA on pooled sera collected at week 11 and tested against *S*. Typhimurium D23580 isolates. Bactericidal activity was determined as serum dilutions necessary to obtain 50% CFU reduction at T180 compared with T0. Bars represent the mean SBA titers ± SEM of triplicate samples. **(C)** Anti-O:4,5 IgG1, IgG2a, IgG2b, and IgG3 assessed by ELISA in individual serum samples collected at week 11. Values are reported as GMT ± SEM. Statistical analysis was performed using one-way ANOVA and Tukey’s post test for multiple comparisons. *P ≤ 0.05, **P ≤ 0.01, and ***P ≤ 0.001.

All monovalent and bivalent, adjuvanted or unadjuvanted, conjugate vaccines elicited levels of anti-O:4,5 IgG serum titers significantly higher (*P* < 0.001) than the control groups unconjugated OAg (O:4,5 or O:9 polysaccharides), saline and CRM_197_ (Figure 2A; Table S1 in Supplementary Material). The bivalent formulation adjuvanted with alum + CpG triggered the highest anti-O:4,5 IgG response, which was significantly higher compared to bivalent vaccine with alum only or unadjuvanted formulations (*P* < 0.05; Figure 2A; Tables S1 and S2 in Supplementary Material). SBA was observed for both monovalent O:4,5-CRM_197_ and bivalent vaccines (both adjuvanted and unadjuvanted) (Figure 2B). On the contrary, no functional activity against *S*. Typhimurium D23580 was observed in mice immunized with Enteritidis O:9-conjugate (Figure 2B), although antibodies cross-reactive with O:4,5 were detected by ELISA (Figure 2A). The analysis of anti-O:4,5 serum IgG subclasses showed a strong predominance of IgG1 in all groups of mice vaccinated with conjugate vaccines suggesting a Th2 polarization (Figure 2C; Table S5 in Supplementary Material). The bivalent vaccine adjuvanted with alum only elicited a stronger Th2 response compared to the same vaccine adjuvanted with alum + CpG, with an IgG1/IgG2a ratio fivefold higher (*P* < 0.01; Table S6 in Supplementary Material).

**Figure 3:**
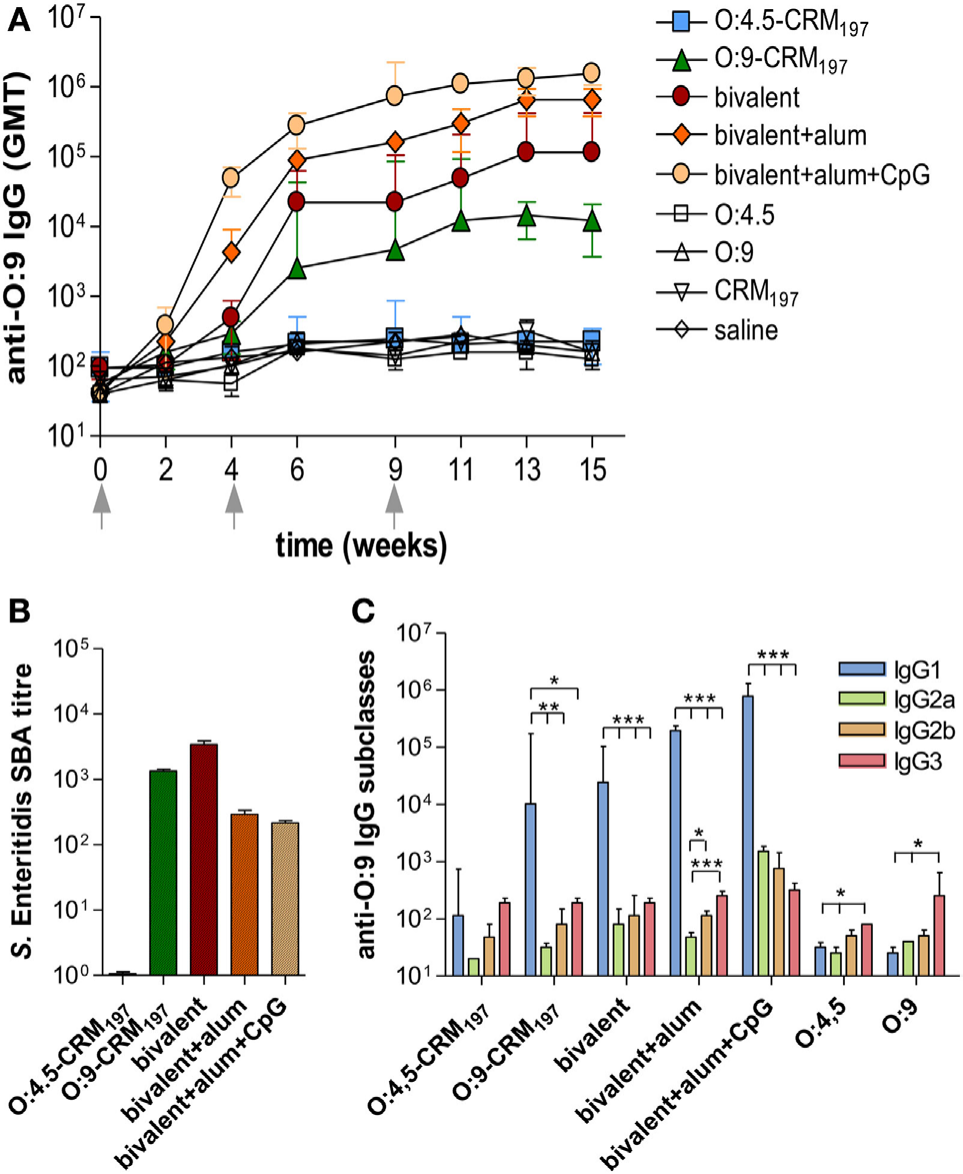
***S*. Enteritidis O-antigen-specific serum IgG, serum bactericidal activity (SBA), and IgG subclasses in CB6F1 mice**. Mice were subcutaneously immunized at weeks 0, 4, and 9 with different vaccine formulations, as reported in Table 2. **(A)** Time course of anti-O:9 IgG assessed by ELISA in individual serum samples on weeks 0, 2, 4, 6, 9, 11, 13, and 15 following the first immunization. Arrows represent the timing of immunizations. Values are reported as GMT ± SEM. **(B)** SBA on pooled sera collected at week 11 and tested against *S*. Enteritidis CMCC4314 isolates. Bactericidal activity was determined as serum dilutions necessary to obtain 50% CFU reduction at T180 compared with T0. Bars represent the mean SBA titers ± SEM of triplicate samples. **(C)** Anti-O:9 IgG1, IgG2a, IgG2b, and IgG3 assessed by ELISA on individual serum samples collected at week 11. Values are reported as GMT ± SEM. Statistical analysis was performed using one-way ANOVA and Tukey’s post test for multiple comparisons. *P ≤ 0.05, **P ≤ 0.01, ***P ≤ 0.001.

The analysis of O:9 IgG response showed significant levels of serum antibodies in mice immunized with the Enteritidis monovalent or bivalent vaccines and, as expected, not in mice immunized with *S*. Typhimurium monovalent vaccine (Figure 3A; *P* < 0.05 compared to control groups; Table S3 in Supplementary Material). As for anti-O:4,5, the bivalent vaccine including alum + CpG induced anti-O:9 IgG serum titers that were significantly higher compared to formulation including alum only or without adjuvants (*P* < 0.05; Figure 3A; Tables S3 and S4 in Supplementary Material). Functional activity against *S*. Enteritidis CMCC4314 was observed for monovalent O:9-CRM_197_ and all bivalent vaccines (Figure 3B). Significantly higher levels of anti-O:9 IgG1 subclass were detected compared to IgG2a, IgG2b, and IgG3 in mice immunized with O:9 monovalent and bivalent conjugate formulations, confirming a Th2 polarization (*P* < 0.05; Figure 3C; Table S7 in Supplementary Material). The bivalent vaccine adjuvanted with alum only elicited a stronger Th2 response compared to the same vaccine adjuvanted with alum + CpG, with an IgG1/IgG2a ratio ninefold higher (*P* < 0.001; Table S8 in Supplementary Material). No statistical differences were observed in the ratio between IgG1 and other IgG subclasses (data not shown).

Data observed in CB6F1 mice were confirmed in C57BL/6 mice immunized according to the experimental design reported in Figure S1A in Supplementary Material and Table 2. As expected, in this mouse strain, known to be less skewed toward a humoral response, antigen-specific antibodies’ titers were lower compared to those of CB6F1 mice. A significant increase in anti-O:4,5 or anti-O:9 IgG was observed in animals immunized with all adjuvanted glycoconjugate formulations with respect to the control group injected with the adjuvant only (*P* < 0.001; Figures S1B,E and Tables S9 and S11 in Supplementary Material). The highest levels of anti-O:4,5 or anti-O:9 IgG were induced after immunization with the vaccines adjuvanted with both alum and CpG compared to the same vaccines adjuvanted with alum only (*P* < 0.05; Tables S10 and S12 in Supplementary Material). SBA was observed in mice immunized with bivalent and homologous monovalent vaccine formulations (Figures S1C,F in Supplementary Material). No SBA against *S*. Typhimurium was observed for monovalent Enteritidis conjugate (Figure S1C in Supplementary Material), although antibodies cross-reactive with O:4,5 were detected by ELISA (Figure S1B in Supplementary Material), confirming data previously observed for CB6F1 mice (Figure 2B).

Similar to what observed with CB6F1 mice, the analysis of IgG subclasses showed that the presence of CpG as adjuvant in vaccine formulations enhanced the anti-O:4,5 and anti-O:9 IgG2b and IgG2c response compared to monovalent or bivalent vaccines adjuvanted with alum only, skewing the response toward a weaker Th2 polarization (Figures S1D,G and Tables S13–S16 in Supplementary Material).

All conjugate vaccines induced anti-CRM_197_-specific serum IgG significantly higher than unconjugated CRM_197_ in CB6F1 mice (*P* < 0.001; Figure S2 in Supplementary Material). The alum plus CpG adjuvanted bivalent formulation elicited a significantly higher response compared to the unadjuvanted bivalent vaccine (Figure S1 in Supplementary Material).

Serum anti-O:4,5 IgM were detected in groups of CB6F1 mice immunized with bivalent or Typhimurium monovalent conjugate, but not with Enteritidis monovalent conjugate, and titers were up to three times higher compared to control groups after the second immunization. No differences were observed for anti-O:9 IgM elicited among all conjugate formulations (data not shown).

Low levels of serum anti-O:4,5 and O:9 IgA were observed in all glycoconjugate formulations tested after two or three immunizations, with no differences compared to control groups (data not shown).

### Local Antibody Responses in CB6F1 Mice Immunized with Adjuvanted and Unadjuvanted Conjugate Vaccines

The local anti-O:4,5 and O:9 antibody response was analyzed in the intestinal tract of CB6F1 mice immunized with monovalent (O:4,5-CRM_197_ or O:9-CRM_197_) and bivalent (O:4,5-CRM_197_ and O:9-CRM_197_) conjugate formulations, unadjuvanted or adjuvanted with alum only or alum + CpG (Table 2), according to the experimental design reported in Figure 1. Antigen-specific IgG and IgA concentrations, normalized to total IgG and IgA, were evaluated in intestinal washes and in fecal samples.

**Figure 4:**
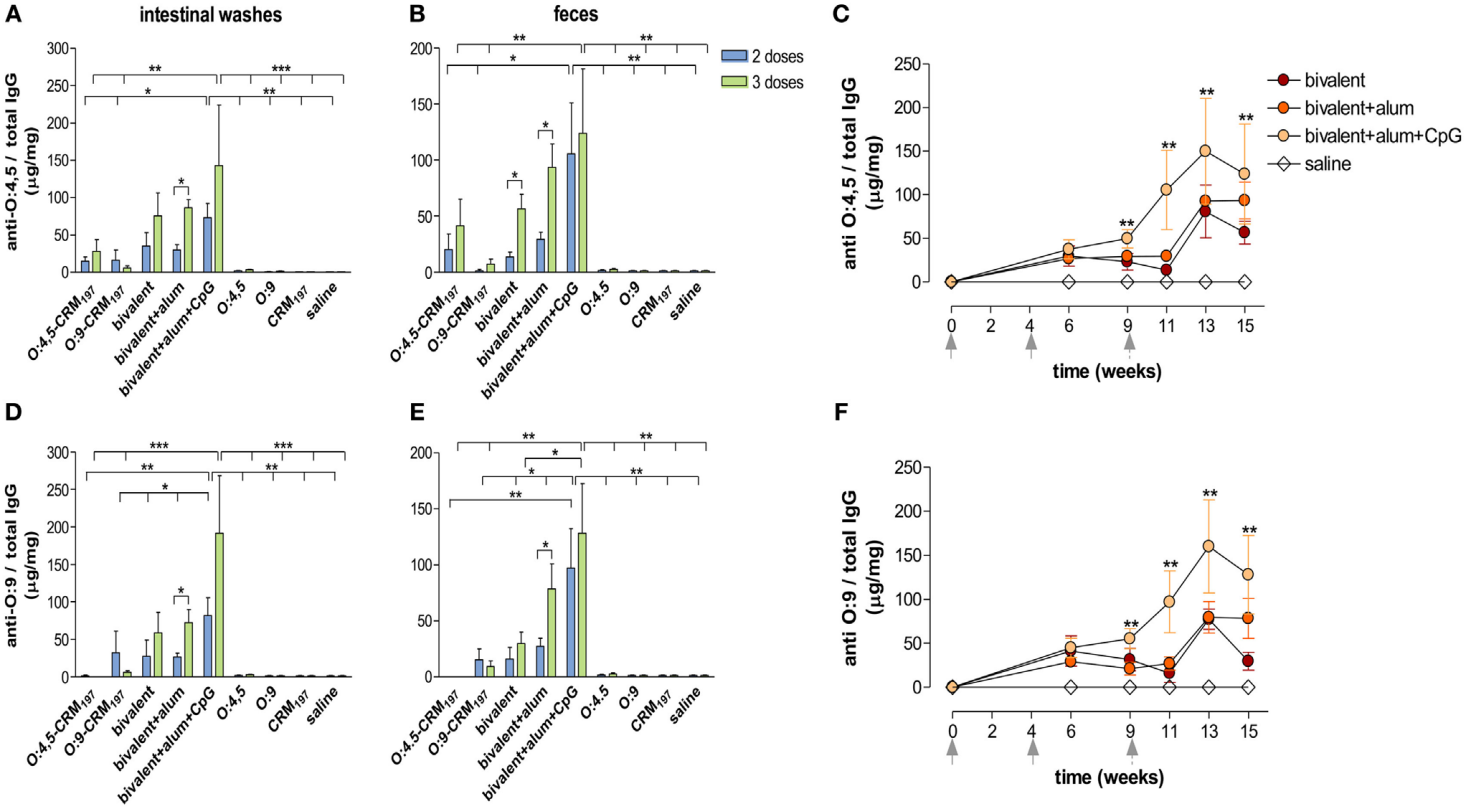
***S*. Typhimurium and Enteritidis O-antigen-specific IgG in intestinal washes and feces**. CB6F1 mice were subcutaneously immunized with different vaccine formulations (as reported in Table 2) at weeks 0, 4, and 9 and sacrificed after two doses (week 11, blue histograms) or three doses (week 15, green histograms). Anti-O:4,5 and anti-O:9 IgG were assessed by ELISA in individual intestinal washes **(A,D)** and fecal samples **(B,E)** collected after two or three immunizations. **(C,F)**. Time course of anti-O:4,5 and anti-O:9 IgG assessed on fecal samples collected on weeks 0, 6, 9, 11, 13, and 15 from mice immunized with adjuvanted or unadjuvanted bivalent vaccines. All values are reported as mean concentration ± SEM. Statistical analysis were performed using one-way ANOVA and Tukey’s post test for multiple comparisons, or two-tailed Student’s *t*-test to compare differences of the same vaccine at different time points. *P ≤ 0.05, **P ≤ 0.01, and ***P ≤ 0.001.

Significantly higher levels of anti-O:4,5 and anti-O:9 IgG were detected in intestinal washes of mice immunized (two or three times) with the bivalent vaccine adjuvanted with alum + CpG, in comparison to unconjugated polysaccharides (*P* < 0.01) and O:4,5 monovalent conjugate vaccines (*P* < 0.05; Figures 4A,D).

In fecal samples, significant anti-O:9 fecal IgG was induced by the bivalent vaccine adjuvanted with alum + CpG with respect to bivalent vaccine adjuvanted with alum only or unadjuvanted (*P* < 0.05) and to monovalent conjugate or unconjugate (*P* < 0.01; Figure 4E) vaccines. Significant levels of anti-O:4,5 fecal IgG were also detected in mice immunized with the bivalent vaccine adjuvanted with alum + CpG, in comparison to unconjugated polysaccharides (*P* < 0.01) and O:4,5 monovalent conjugate vaccines (*P* < 0.05; Figure 4B).

The time course analysis showed that the increase in fecal anti-O:4,5 and anti-O:9 IgG, induced by the bivalent vaccine adjuvanted with alum + CpG, started at week 9 with a peak 4 weeks after the third immunization (week 13), and levels were maintained also at week 15 (Figures 4C,F).

Interestingly, a positive correlation of both anti-O:4,5 and anti-O:9 IgG levels was found between intestinal washes and fecal samples for each mouse with *r* ≥ 0.93 (*P* ≤ 0.05) in mice immunized with the bivalent vaccine adjuvanted with alum + CpG (data not shown). In the same group, positive correlation was also observed between sera and both intestinal washes and fecal samples with *r* ≥ 0.92 (*P* ≤ 0.05) and *r* ≥ 0.72 (*P* ≤ 0.05), respectively (data not shown).

Very low levels of both anti-O:4,5 and anti-O:9 IgA, normalized to total IgA, were observed in intestinal washes and fecal samples of mice immunized (two or three times) with all conjugate formulations, and no differences were detected compared to control group (data not shown).

### Cellular Immune Response in CB6F1 Mice Immunized with Adjuvanted and Unadjuvanted Conjugate Vaccines

**Figure 5:**
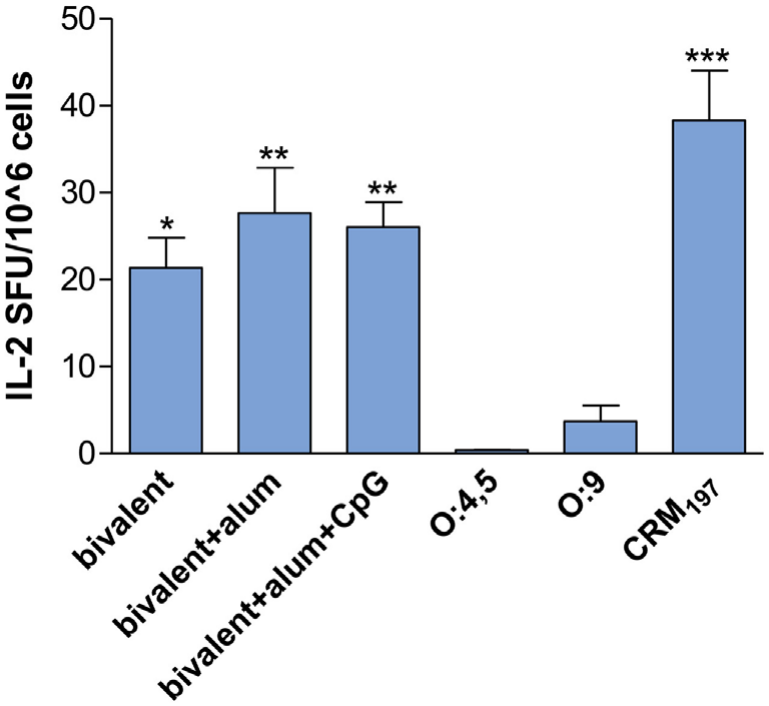
**IL-2 splenocyte response**. CB6F1 mice were subcutaneously immunized at weeks 0 and 4 with different vaccine formulations, as reported in Table 2. Splenocytes were collected at week 11 following the first immunization, and IL-2 production was evaluated by ELISPOT assay. The number of antigen-specific spot-forming units (SFU) of CRM_197_ *in vitro* stimulated *minus* the respective unstimulated samples is shown. Each bar represents the mean number of SFU/10^6^ cells of pooled splenocytes. Statistical analysis was performed using one-way ANOVA and Tukey’s post test for multiple comparisons. *P ≤ 0.05, **P ≤ 0.01, and ***P ≤ 0.001 *versus* both unconjugated polysaccharides.

IL-2 production was investigated in splenocytes of CB6F1 mice after *in vitro* restimulation with the carrier protein CRM_197_. Bivalent conjugate vaccines were more efficient in stimulating a T-cell response, in terms of IL-2 SFU, compared to control groups vaccinated with unconjugated O:4,5 and O:9 (*P* < 0.05; Figure 5)_._ Moreover, the presence of adjuvants in the bivalent vaccine formulations induced a higher number of IL-2 producing cells (*P* < 0.01, Figure 5). IL-2 SFU similar to the control groups or saline (≤ 2 SFU/10^6^ cells, data not shown) were detected in all glycoconjugate immunized groups after *in vitro* restimulation with OAg (data not shown). No increase in IFN-γ SFU was detected in all groups, independently of the antigen used for the *in vitro* stimulation (data not shown). As expected, immunization of mice with each unconjugated OAg did not induce appreciable production of cytokines, being a T-cell independent antigen.

## Discussion

In this work, we have evaluated the immunogenicity of monovalent and bivalent glycoconjugates as promising candidate vaccines against *S*. Typhimurium and *S*. Enteritidis. A bivalent conjugate vaccine against both *S*. Typhimurium and *S*. Enteritidis would be a promising tool to overcome the frequent co-endemicity of these serogroups. The conjugation of a polysaccharide antigen to a carrier protein constitutes a strategy of proven effectiveness to enhance the immunogenicity of polysaccharide alone, by inducing T helper cell-mediated antibody responses. Additionally, the use of adjuvants can achieve qualitative alteration of the immune response, improve vaccine efficacy, and shape the immune response (16, 17).

In previous work, we undertook an extensive strain selection process, which resulted in the identification of *S*. Typhimurium and *S*. Enteritidis isolates that could be used as a safe and effective source for glycoconjugate vaccines against iNTS disease in Africa (14). Those conjugates were able to induce high antibody levels with high breadth of serovar-specific strain coverage, when tested as monovalent unadjuvanted vaccines. Here, we employed those selected glycoconjugates to further investigate the humoral and cellular, systemic and local, immune responses elicited in bivalent formulations, adjuvanted or not.

In the present study, testing monovalent and bivalent glycoconjugates against *S*. Typhimurium and *S*. Enteritidis in two mouse strains, we have shown: (i) increased magnitude and persistence of anti-O:4,5 and anti-O:9 IgG in mice vaccinated with bivalent or homologous monovalent conjugate vaccines compared to unconjugated OAg; (ii) no interference in IgG response when both *S*. Typhimurium and *S*. Enteritidis conjugates were administered together as a bivalent formulation; (iii) higher IgG levels in mice immunized with bivalent vaccine adjuvanted with alum only, or with alum + CpG, compared to unadjuvanted vaccine; (iv) SBA with bivalent and homologous monovalent vaccine formulations; (v) prevalence of specific IgG1 subclass for monovalent and bivalent conjugate vaccines; (vi) higher ratio of IgG1/IgG2a for vaccine adjuvanted with alum compared to alum + CpG (likely reflecting the influence of these adjuvants on the Th response); (vii) presence of IgG in intestinal washes and fecal samples induced by alum + CpG-adjuvanted bivalent vaccines; (viii) positive correlation of IgG levels between sera, intestinal washes, and fecal samples; and (ix) higher IL-2 production in splenocytes of mice vaccinated with adjuvanted bivalent conjugate vaccines.

Both *S*. Typhimurium and *S*. Enteritidis monovalent or bivalent vaccine formulations were highly immunogenic in both CB6F1 and C57BL/6 mouse strains. The *S*. Typhimurium conjugate elicited an anti-O:4,5-specific IgG response and no cross-reaction with *S*. Enteritidis O:9 antigen. In contrast, the *S*. Enteritidis conjugate surprisingly was able to trigger an IgG response that cross-reacted with *S*. Typhimurium OAg, reaching serum titers as high as those induced by the equivalent *S*. Typhimurium conjugate. The reason for such cross-reactivity is under investigation, but is most likely due to antibody directed against common OAg epitopes, such as O:1 and O:12, or against the common core region. These more internal antigens may become accessible to antibody binding when coated on an ELISA plate, while remaining less accessible in live bacteria, thus explaining the lack of functional bactericidal activity of antibody directed against them. In earlier studies, it was found that monoclonal IgG3 antibodies directed against O:4 and O:12 showed a marked difference in protective activity, with the anti-O:12 monoclonal 2,500-fold less protective than the O:4 monoclonal (40).

Regarding IgG subclass switch, it is commonly accepted that Th1/Th2 polarization is dependent on different factors, such as the genetic predisposition of mouse strain, the vaccine formulation used, and the route of immunization. Adjuvants play a critical role in shaping the immune response to vaccine formulations. No study has been conducted so far using adjuvanted OAg conjugates with either monovalent or bivalent iNTS conjugate formulations. Alum (aluminum salts) is known to induce a Th2 response in mice (41) but a mixture of Th2 and Th1 in humans (42), while CpG ODN activates antigen-presenting cells and B cells by binding to Toll-like receptor 9 (TLR9). The interaction of TLR9 with CpG motifs initiates a cascade of events resulting in the secretion of Th1-type cytokines, and antibody response is most efficient when the CpG is chemically linked (43) or associated with the antigen and bound to alum (44), for a synergic effect (45). A Th1/Th2 response can influence the IgG subclasses that are produced, which in turn can influence the bactericidal activity of the sera from immunized mice (12). Evaluating whether formulation with Th1/Th2 adjuvants can improve the anti-OAg response and the bactericidal activity can potentially guide optimal conjugate formulation.

In this work, both alum and CpG adjuvants administered with conjugate formulations modulated the skewing of the immune response, confirming what has been previously reported in relation to IgG subclasses switching (46). More specifically, our data demonstrate the ability of glycoconjugate vaccines to drive a Th2 immune response, as indicated by the dominant IgG1 response, particularly with the alum-adjuvanted vaccines. The adjuvant CpG, combined with alum, was able to reduce the Th2/Th1 ratio in both CB6F1 and C57BL/6 mice. The presence of CpG in the vaccine formulation enhances the IgG2a, IgG2b, and IgG2c titers compared to alum only, in monovalent and bivalent conjugate vaccines.

In terms of CRM_197_-specific serum IgG, all conjugates were able to generate much higher responses than the group immunized with unconjugated CRM_197_. Indeed, mice injected twice with unconjugated CRM_197_ were not able to produce high levels of CRM_197_-specific IgG. Similar data were previously obtained using Vi-CRM_197_, a *Salmonella* Typhi conjugate vaccine (47), but are actually discordant from what has been published by Pecetta et al. (48), who found higher anti-CRM_197_ responses induced by the protein alone compared to the protein conjugated to meningococcal polysaccharide. In this specific case, the authors postulated that structural modifications of the carrier protein during the glycoconjugation induced conformational changes with consequent alteration of the antibody recognition and modulation of immunogenicity. However, they also found lower intrinsic immunogenicity of CRM_197_ compared to diphtheria toxoid, which could be related to a different stability of the two proteins. In our case, we postulate that the conjugation of OAg chains to CRM_197_ leads to a “stabilization” of the protein, enhancing its immunogenicity.

Here, we have shown that the bivalent conjugate vaccine adjuvanted with alum + CpG triggered the highest anti-O:4,5 and anti-O:9 IgG in intestinal tract, with statistically significant levels in intestinal washes and fecal samples compared to the respective monovalent conjugate and unconjugate formulations. A positive correlation was observed between antigen-specific IgG detected in intestinal washes collected when mice were sacrificed, and fecal samples collected at the same time point, suggesting that feces constitute valid alternative samples to intestinal washes to follow the intestinal immune response *in vivo*. Good correlation was also observed for IgG levels between serum and intestinal washes and between serum and fecal samples, confirming what was previously found with Vi-CRM_197_ vaccine (47). These findings support the concept of passive diffusion of IgG from blood to intestine and confirm the potential of parenteral immunization in inducing antibody response in local compartments, as reported in different works using other conjugate vaccines (47), soluble immunogens (49), or purified flagellin (50). The higher systemic and local antibody response obtained for bivalent adjuvanted conjugate vaccines reflected the higher T cell response observed in terms of IL-2 SFU in the spleen. These data are consistent with the critical involvement of the pure T-dependent antigen CRM_197_ for the *in vitro* cytokine response, as confirmed by the absence of IL-2 production after restimulation with each unconjugated polysaccharide.

Taken together, these data demonstrate the ability of monovalent and bivalent conjugate vaccines against *S*. Typhimurium and *S*. Enteritidis to induce systemic and local immune responses, and highlight the suitability of the bivalent glycoconjugate formulation, especially adjuvanted with alum + CpG, as a promising candidate vaccine against iNTS disease.

## Author Contributions

FF, SR, FMi, CM, and DM conceived and designed the experiments. FF, FMi, LL, RA, and FMa performed the experiments. FF, SR, FMi, RA, FMa, CM, and DM analyzed the data. FF, SR, FMi, and DM wrote the paper. SR, FMi, LL, RA, and CM provided reagents. FF, SR, FMi, LL, RA, FMa, CM, and DM revised the manuscript, read, and approved the final manuscript.

## Conflict of Interest Statement

All authors have declared the following interests: SR, FMi, LL, RA, and FMa were employees of the Novartis Vaccines Institute for Global Health (NVGH) at the time of the study. Following the acquisition of Novartis Vaccines & Diagnostics and NVGH by the GSK group of companies in March 2015, SR, FMi, LL, RA, and FMa are now employees of the GSK Vaccines Institute for Global Health (GVGH), part of the GSK group of companies. CM was employed by NVGH from September 2010 to December 2014 and held a Clinical Research Fellowship from GSK (2008–2015) through the University of Birmingham during the course of the study. FF and DM report no financial conflicts of interest.

## Supplementary Material

Figure S1 ***S*. Typhimurium and *S*. Enteritidis O-antigen-specific serum IgG, serum bactericidal activity (SBA), and IgG subclasses in C57BL/6 mice**. Mice were subcutaneously immunized at weeks 0 and 4 with different vaccine formulations, as reported in Table 2. **(A)** Experimental design for immunogenicity study. **(B,E)** Time course of anti-O:4,5 **(B)** and anti-O:9 **(E)** IgG assessed by enzyme-linked immunosorbent assay (ELISA) in individual serum samples on weeks 0, 2, 4, 6, and 7 following the first immunization. Arrows represent the timing of immunizations. **(C,F)** SBA on pooled sera collected at week 7 and tested against *S*. Typhimurium D23580 **(C)** and *S*. Enteritidis CMCC4314 **(F)** isolates. Bactericidal activity was determined as serum dilutions necessary to obtain 50% percent colony-forming unit reduction at T180 compared with T0. Bars represent the mean SBA titers ± SEM of triplicate samples. **(D,G)** Anti-O:4,5 **(D)** and anti-O:9 **(G)** IgG1, IgG2b, IgG2c, and IgG3 were assessed by ELISA on individual serum samples collected at week 7. All values are reported as geometric mean titers (GMT) ± SEM. Statistical analysis was performed using one-way analysis of variance and Tukey’s post test for multiple comparisons. **P* ≤ 0.05, ***P* ≤ 0.01, and ****P* ≤ 0.001.

Figure S2 **CRM197-specific serum IgG response**. CB6F1 mice were subcutaneously immunized at weeks 0 and 4 with different vaccine formulations, as reported in Table 2. CRM_197_-specific IgG was assessed by ELISA in individual serum samples collected at week 11. Values are reported as geometric mean titers (GMT) ± SEM. One-way analysis of variance and Tukey’s post test for multiple comparisons were used for comparing antibody response between different groups. ****P* ≤ 0.001 versus control groups.
