# Supplementary figures and images for: Immunogenicity of a Bivalent Adjuvanted Glycoconjugate Vaccine against Salmonella Typhimurium and Salmonella Enteritidis
Source: Front Immunol. 2017 Feb 27;8:168. doi: 10.3389/fimmu.2017.00168 (PMC5326758; doi:10.3389/fimmu.2017.00168)

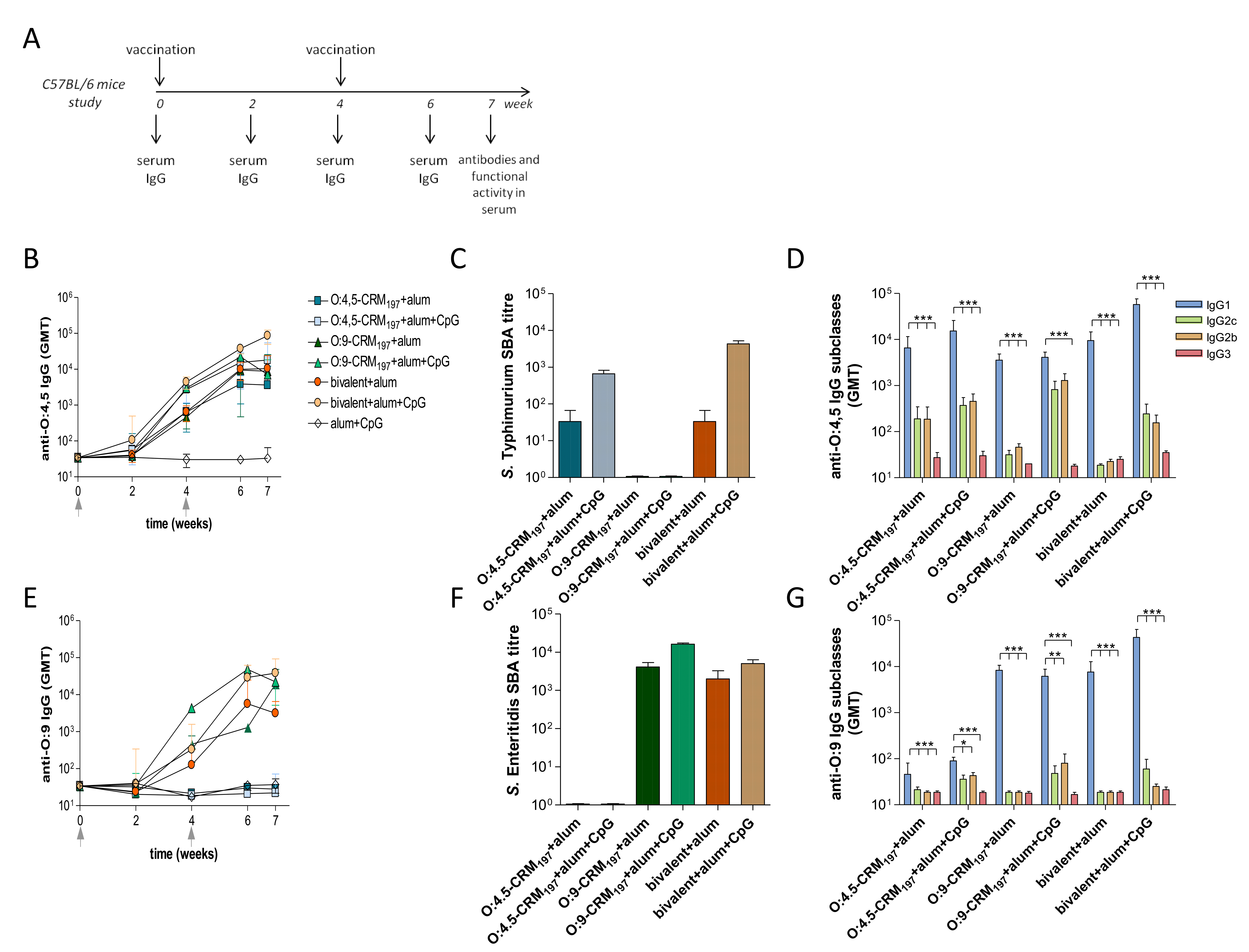

Supplement: Figure S1 — S. Typhimurium and S. Enteritidis O-antigen-specific serum IgG, serum bactericidal activity (SBA), and IgG subclasses in C57BL/6 mice. Mice were subcutaneously immunized at weeks 0 and 4 with different vaccine formulations, as reported in Table 2. (A) Experimental design for immunogenicity study. (B,E) Time course of anti-O:4,5 (B) and anti-O:9 (E) IgG assessed by enzyme-linked immunosorbent assay (ELISA) in individual serum samples on weeks 0, 2, 4, 6, and 7 following the first immunization. Arrows represent the timing of immunizations. (C,F) SBA on pooled sera collected at week 7 and tested against S. Typhimurium D23580 (C) and S. Enteritidis CMCC4314 (F) isolates. Bactericidal activity was determined as serum dilutions necessary to obtain 50% percent colony-forming unit reduction at T180 compared with T0. Bars represent the mean SBA titers ± SEM of triplicate samples. (D,G) Anti-O:4,5 (D) and anti-O:9 (G) IgG1, IgG2b, IgG2c, and IgG3 were assessed by ELISA on individual serum samples collected at week 7. All values are reported as geometric mean titers (GMT) ± SEM. Statistical analysis was performed using one-way analysis of variance and Tukey’s post test for multiple comparisons. *P ≤ 0.05, **P ≤ 0.01, and ***P ≤ 0.001. [file image_1.tif]

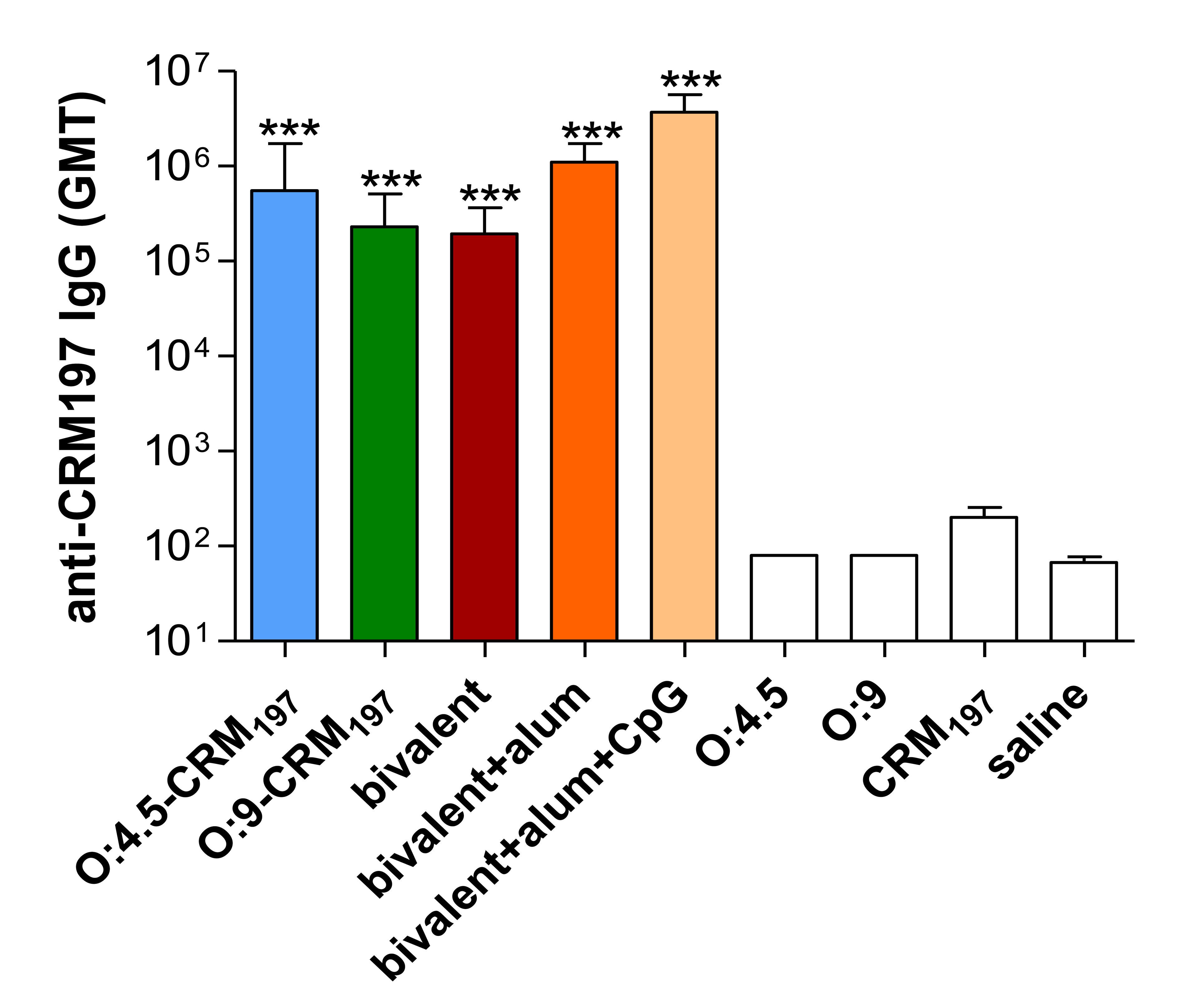

Supplement: Figure S2 — CRM197-specific serum IgG response. CB6F1 mice were subcutaneously immunized at weeks 0 and 4 with different vaccine formulations, as reported in Table 2. CRM197-specific IgG was assessed by ELISA in individual serum samples collected at week 11. Values are reported as geometric mean titers (GMT) ± SEM. One-way analysis of variance and Tukey’s post test for multiple comparisons were used for comparing antibody response between different groups. ***P ≤ 0.001 versus control groups. [file image_2.tif]
