# Supplementary material for: Immunogenicity of a Bivalent Adjuvanted Glycoconjugate Vaccine against Salmonella Typhimurium and Salmonella Enteritidis
Source: Front Immunol. 2017 Feb 27;8:168. doi: 10.3389/fimmu.2017.00168 (PMC5326758; doi:10.3389/fimmu.2017.00168)
Supplement: Supplementary file 3 [file table_1.pdf]

Table S1. Statistical significance of O:4,5 – specific serum IgG titers in CB6F1 mice.

group [a]	*versus*	*P* values [b]						
		week 2	week 4	week 6	week 9	week 11	week 13	week 15
bivalent $+Al^{3+}$ + CpG	O:4,5-CRM_{197}	**<0.001**	**<0.001**	**<0.05**	**<0.05**	**<0.01**	**<0.05**	**<0.05**
	O:9-CRM_{197}	**<0.001**	**<0.001**	**<0.001**	**<0.001**	**<0.001**	**<0.001**	**<0.001**
	bivalent	**<0.001**	**<0.001**	**<0.05**	**<0.01**	**<0.05**	>0.05	>0.05
	bivalent$+Al^{3+}$	**<0.01**	**<0.001**	**<0.001**	**<0.001**	**<0.001**	**<0.001**	**<0.001**
	O:4,5	**<0.001**	**<0.001**	**<0.001**	**<0.001**	**<0.001**	**<0.001**	**<0.001**
	O:9	**<0.001**	**<0.001**	**<0.001**	**<0.001**	**<0.001**	**<0.001**	**<0.001**
	CRM_{197}	**<0.001**	**<0.001**	**<0.001**	**<0.001**	**<0.001**	**<0.001**	**<0.001**
	saline	**<0.001**	**< 0.001**	**<0.001**	**<0.001**	**<0.001**	**<0.001**	**<0.001**
bivalent $+Al^{3+}$	O:4,5-CRM_{197}	>0.05	>0.05	>0.05	>0.05	>0.05	>0.05	>0.05
	O:9-CRM_{197}	>0.05	**< 0.001**	**<0.01**	**< 0.001**	**<0.001**	**< 0.001**	**<0.001**
	bivalent	>0.05	**<0.001**	>0.05	>0.05	>0.05	>0.05	**<0.001**
	O:4,5	**<0.05**	**<0.001**	**<0.001**	**<0.001**	**<0.001**	**<0.001**	**<0.001**
	O:9	**<0.05**	**<0.001**	**<0.001**	**<0.001**	**<0.001**	**<0.001**	**<0.001**
	CRM_{197}	**<0.01**	**<0.001**	**<0.001**	**<0.001**	**<0.001**	**<0.001**	**<0.001**
	saline	**<0.01**	**<0.001**	**<0.001**	**<0.001**	**<0.001**	**<0.001**	**<0.001**
bivalent	O:4,5-CRM_{197}	>0.05	>0.05	>0.05	>0.05	>0.05	>0.05	>0.05
	O:9-CRM_{197}	>0.05	>0.05	>0.05	>0.05	**<0.05**	**<0.05**	**<0.01**
	O:4,5	>0.05	>0.05	**<0.001**	**<0.001**	**<0.001**	**<0.001**	**<0.001**
	O:9	>0.05	>0.05	**<0.001**	**<0.001**	**<0.001**	**<0.001**	**<0.001**
	CRM_{197}	>0.05	>0.05	**<0.001**	**<0.001**	**<0.001**	**<0.001**	**<0.001**
	saline	>0.05	>0.05	**<0.001**	**<0.001**	**<0.001**	**<0.001**	**<0.001**
O:9-CRM_{197}	O:4,5-CRM_{197}	>0.05	>0.05	>0.05	>0.05	>0.05	>0.05	>0.05
	O:4,5	>0.05	>0.05	>0.05	**< 0.05**	**<0.001**	**<0.001**	**<0.001**
	O:9	>0.05	>0.05	>0.05	>0.05	**<0.001**	**<0.01**	**<0.001**
	CRM_{197}	>0.05	>0.05	>0.05	**< 0.05**	**<0.001**	**<0.01**	**<0.001**
	saline	>0.05	>0.05	>0.05	**< 0.05**	**<0.001**	**<0.001**	**<0.001**
O:4,5-CRM_{197}	O:4,5	>0.05	**<0.05**	**<0.001**	**<0.001**	**<0.001**	**<0.001**	**<0.001**
	O:9	>0.05	**<0.05**	**<0.001**	**<0.001**	**<0.001**	**<0.001**	**<0.001**
	CRM_{197}	>0.05	>0.05	**<0.001**	**<0.001**	**<0.001**	**<0.001**	**<0.001**
	saline	>0.05	>0.05	**<0.001**	**<0.001**	**<0.001**	**<0.001**	**<0.001**

[a] O:4,5-CRM_{197}, *S.* Typhimurium O-antigen conjugated with CRM_{197}; O:9-CRM_{197}, *S.* Enteritidis O-antigen conjugated with CRM_{197}; bivalent, O:4,5-CRM_{197} + O:9-CRM_{197}; bivalent + Al^{3+}, O:4,5-CRM_{197} + O:9-CRM_{197} + Al^{3+}; bivalent + Al^{3+} + CpG, O:4,5-CRM_{197} + O:9-CRM_{197} + Al^{3+} + CpG; O:4,5, *S.* Typhimurium O-antigen; O:9, *S.* Enteritidis O-antigen; CRM_{197}: non-toxic mutant of diphtheria toxin.

[b] *P* values assessed using one-way analysis of variance (ANOVA) and Tukey's post test for multiple comparisons.

Table S2. Statistical significance of O:4,5 – specific serum IgG titers in CB6F1 mice.

		P values						
**group** [a]	***versus***	**week 2**	**week 4**	**week 6**	**week 9**	**week 11**	**week 13**	**week 15**
**bivalent+Al^{3+}+CpG**	**bivalent+Al^{3+}**	**0.0188**	**0.0007**	**0.0005**	**<0.0001**	0.1466	0.134	0.121

[a] bivalent + Al^{3+}, O:4,5-CRM_{197} + O:9-CRM_{197} + Al^{3+}; bivalent + Al^{3+} + CpG, O:4,5-CRM_{197} + O:9-CRM_{197} + Al^{3+} + CpG.

[b] *P* values assessed using two tailed Student t test.

Table S3. Statistical significance of O:9 – specific serum IgG titers in CB6F1 mice.

group [a]	*versus*	*P* values [b]						
		week 2	week 4	week 6	week 9	week 11	week 13	week 15
bivalent +Al^{3+} + CpG	O:4,5-CRM$_{197}$	**<0.01**	**<0.01**	**<0.001**	**<0.001**	**<0.001**	**<0.001**	**<0.001**
	O:9-CRM$_{197}$	>0.05	>0.05	**<0.001**	**<0.001**	**<0.001**	**<0.001**	**<0.001**
	bivalent	**<0.05**	**<0.05**	**<0.05**	**<0.01**	**<0.001**	**<0.05**	**<0.05**
	bivalent+Al^{3+}	>0.05	>0.05	>0.05	>0.05	>0.05	>0.05	>0.05
	O:4,5	**<0.001**	**<0.001**	**<0.001**	**<0.001**	**<0.001**	**<0.001**	**<0.001**
	O:9	**<0.001**	**<0.001**	**<0.001**	**<0.001**	**<0.001**	**<0.001**	**<0.001**
	CRM$_{197}$	**<0.01**	**<0.01**	**<0.001**	**<0.001**	**<0.001**	**<0.001**	**<0.001**
	saline	**<0.001**	**<0.001**	**<0.001**	**<0.001**	**<0.001**	**< 0.001**	**<0.001**
bivalent +Al^{3+}	O:4,5-CRM$_{197}$	>0.05	>0.05	**<0.001**	**<0.001**	**<0.001**	**<0.001**	**<0.001**
	O:9-CRM$_{197}$	>0.05	>0.05	**<0.001**	**<0.01**	**<0.001**	**<0.001**	**<0.001**
	bivalent	>0.05	>0.05	>0.05	>0.05	>0.05	>0.05	>0.05
	O:4,5	**<0.05**	**<0.05**	**<0.001**	**<0.001**	**<0.001**	**<0.001**	**<0.001**
	O:9	**<0.05**	**<0.05**	**<0.001**	**<0.001**	**<0.001**	**<0.001**	**<0.001**
	CRM$_{197}$	>0.05	>0.05	**<0.001**	**<0.001**	**<0.001**	**<0.001**	**<0.001**
	saline	>0.05	>0.05	**<0.001**	**<0.001**	**<0.001**	**<0.001**	**<0.001**
bivalent	O:4,5-CRM$_{197}$	>0.05	>0.05	**<0.001**	**<0.001**	**<0.001**	**<0.001**	**<0.001**
	O:9-CRM$_{197}$	>0.05	>0.05	>0.05	>0.05	>0.05	>0.05	>0.05
	O:4,5	>0.05	>0.05	**<0.001**	**<0.001**	**<0.001**	**<0.001**	**<0.001**
	O:9	>0.05	>0.05	**<0.001**	**<0.001**	**<0.001**	**<0.001**	**<0.001**
	CRM$_{197}$	>0.05	>0.05	**<0.001**	**<0.001**	**<0.001**	**<0.001**	**<0.001**
	saline	>0.05	>0.05	**<0.001**	**<0.001**	**<0.001**	**<0.001**	**<0.001**
O:9-CRM$_{197}$	O:4,5-CRM$_{197}$	>0.05	>0.05	**<0.05**	**<0.05**	**<0.001**	**<0.001**	**<0.001**
	O:4,5	>0.05	>0.05	>0.05	**<0.01**	**<0.001**	**<0.001**	**<0.001**
	O:9	>0.05	>0.05	>0.05	**<0.05**	**<0.001**	**<0.001**	**<0.001**
	CRM$_{197}$	>0.05	>0.05	**<0.05**	**<0.01**	**< 0.001**	**<0.01**	**<0.001**
	saline	>0.05	>0.05	**<0.05**	**<0.05**	**< 0.001**	**<0.001**	**<0.001**
O:4,5-CRM$_{197}$	O:4,5	>0.05	>0.05	>0.05	>0.05	>0.05	>0.05	>0.05
	O:9	>0.05	>0.05	>0.05	>0.05	>0.05	>0.05	>0.05
	CRM$_{197}$	>0.05	>0.05	>0.05	>0.05	>0.05	>0.05	>0.05
	saline	>0.05	>0.05	>0.05	>0.05	>0.05	>0.05	>0.05

[a] O:4,5-CRM$_{197}$, *S.* Typhimurium O-antigen conjugated with CRM$_{197}$; O:9-CRM$_{197}$, *S.* Enteritidis O-antigen conjugated with CRM$_{197}$; bivalent, O:4,5-CRM$_{197}$ + O:9-CRM$_{197}$; bivalent + Al^{3+}, O:4,5-CRM$_{197}$ + O:9-CRM$_{197}$ + Al^{3+}; bivalent + Al^{3+} + CpG, O:4,5-CRM$_{197}$ + O:9-CRM$_{197}$ + Al^{3+} + CpG; O:4,5, *S.* Typhimurium O-antigen; O:9, *S.* Enteritidis O-antigen; CRM$_{197}$: non-toxic mutant of diphtheria toxin.

[b] *P* values assessed using one-way analysis of variance (ANOVA) and Tukey's post test for multiple comparisons.

Table S4. Statistical significance of O:9 – specific serum IgG titers in CB6F1 mice.

		P values						
**group** [a]	***versus***	**week 2**	**week 4**	**week 6**	**week 9**	**week 11**	**week 13**	**week 15**
**bivalent+Al^{3+}+CpG**	**bivalent+Al^{3+}**	0.3343	**0.0003**	**0.0095**	**0.0116**	0.1428	0.3559	0.1936

[a] bivalent + Al^{3+}, O:4,5-CRM_{197} + O:9-CRM_{197} + Al^{3+}; bivalent + Al^{3+} + CpG, O:4,5-CRM_{197} + O:9-CRM_{197} + Al^{3+} + CpG.

[b] *P* values assessed using two tailed Student t test.

Table S5. Statistical significance of O:4,5 – specific serum IgG1, IgG2a, IgG2b, IgG3 titers in CB6F1 mice.

group [a]	*versus*	*P* values [b]			
		IgG1	IgG2a	IgG2b	IgG3
**bivalent +Al^{3+} + CpG**	**O:4,5-CRM$_{197}$**	>0.05	**<0.01**	>0.05	**<0.01**
	**O:9-CRM$_{197}$**	**<0.01**	**<0.01**	**<0.01**	**<0.01**
	**bivalent**	>0.05	>0.05	>0.05	**<0.05**
	**bivalent+Al^{3+}**	>0.05	**<0.001**	>0.05	>0.05
	**O:4,5**	**<0.001**	**<0.001**	**<0.001**	**<0.001**
	**O:9**	**<0.001**	**<0.001**	**<0.001**	**<0.001**
	**CRM$_{197}$**	**<0.001**	**<0.001**	**<0.001**	**<0.001**
	**saline**	**<0.001**	**<0.001**	**<0.001**	**<0.001**
**bivalent +Al^{3+}**	**O:4,5-CRM$_{197}$**	>0.05	>0.05	>0.05	>0.05
	**O:9-CRM$_{197}$**	>0.05	>0.05	>0.05	>0.05
	**bivalent**	>0.05	>0.05	>0.05	>0.05
	**O:4,5**	**<0.001**	>0.05	>0.05	>0.05
	**O:9**	**<0.001**	>0.05	>0.05	>0.05
	**CRM$_{197}$**	**<0.001**	>0.05	>0.05	>0.05
	**saline**	**<0.001**	>0.05	>0.05	>0.05
**bivalent**	**O:4,5-CRM$_{197}$**	>0.05	>0.05	>0.05	>0.05
	**O:9-CRM$_{197}$**	>0.05	>0.05	>0.05	>0.05
	**O:4,5**	**<0.001**	>0.05	>0.05	>0.05
	**O:9**	**<0.001**	>0.05	>0.05	>0.05
	**CRM$_{197}$**	**<0.001**	**<0.05**	>0.05	>0.05
	**saline**	**<0.001**	**<0.05**	>0.05	>0.05
**O:9-CRM$_{197}$**	**O:4,5-CRM$_{197}$**	>0.05	>0.05	>0.05	>0.05
	**O:4,5**	**<0.05**	>0.05	>0.05	>0.05
	**O:9**	**<0.01**	>0.05	>0.05	>0.05
	**CRM$_{197}$**	**<0.05**	>0.05	>0.05	>0.05
	**saline**	**<0.01**	>0.05	>0.05	>0.05
**O:4,5-CRM$_{197}$**	**O:4,5**	**<0.001**	>0.05	>0.05	>0.05
	**O:9**	**<0.001**	>0.05	>0.05	>0.05
	**CRM$_{197}$**	**<0.01**	>0.05	>0.05	>0.05
	**saline**	**<0.001**	>0.05	>0.05	>0.05

[a] O:4,5-CRM$_{197}$, *S.* Typhimurium O-antigen conjugated with CRM$_{197}$; O:9-CRM$_{197}$, *S.* Enteritidis O-antigen conjugated with CRM$_{197}$; bivalent, O:4,5-CRM$_{197}$ + O:9-CRM$_{197}$; bivalent + Al^{3+}, O:4,5-CRM$_{197}$ + O:9-CRM$_{197}$ + Al^{3+}; bivalent + Al^{3+} + CpG, O:4,5-CRM$_{197}$ + O:9-CRM$_{197}$ + Al^{3+} + CpG; O:4,5, *S.* Typhimurium O-antigen; O:9, *S.* Enteritidis O-antigen; CRM$_{197}$: non-toxic mutant of diphtheria toxin.

[b] *P* values assessed using one-way analysis of variance (ANOVA) and Tukey's post test for multiple comparisons.

Table S6. Statistical significance of O:4,5 – specific serum IgG1, IgG2a, IgG2b, IgG3 titers in CB6F1 mice.

		***P* values [b]**			
**group [a]**	***versus***	**IgG1**	**IgG2a**	**IgG2b**	**IgG3**
**bivalent+Al^{3+}+CpG**	**bivalent+Al^{3+}**	**0.0102**	**< 0.0001**	0.0701	**0.0106**

[a] bivalent + Al^{3+}, O:4,5-CRM_{197} + O:9-CRM_{197} + Al^{3+}; bivalent + Al^{3+} + CpG, O:4,5-CRM_{197} + O:9-CRM_{197} + Al^{3+} + CpG.

[b] *P* values assessed using two tailed Student t test.

Table S7. Statistical significance of O:9 – specific serum IgG1, IgG2a, IgG2b, IgG3 titers in CB6F1 mice.

		P values [b]			
**group** [a]	***versus***	**IgG1**	**IgG2a**	**IgG2b**	**IgG3**
**bivalent $+Al^{3+}$ + CpG**	**O:4,5-CRM_{197}**	**<0.001**	**<0.001**	**<0.01**	>0.05
	**O:9-CRM_{197}**	**<0.05**	**<0.001**	**<0.05**	>0.05
	**bivalent**	>0.05	**<0.001**	>0.05	>0.05
	**bivalent$+Al^{3+}$**	>0.05	**<0.001**	>0.05	>0.05
	**O:4,5**	**<0.001**	**<0.001**	**<0.001**	**<0.001**
	**O:9**	**<0.001**	**<0.001**	**<0.001**	**<0.001**
	**CRM_{197}**	**<0.001**	**<0.001**	**<0.001**	**<0.001**
	**saline**	**<0.001**	**<0.001**	**<0.001**	**<0.001**
**bivalent $+Al^{3+}$**	**O:4,5-CRM_{197}**	**<0.001**	>0.05	>0.05	>0.05
	**O:9-CRM_{197}**	>0.05	>0.05	>0.05	>0.05
	**bivalent**	>0.05	>0.05	>0.05	>0.05
	**O:4,5**	**<0.001**	>0.05	>0.05	>0.05
	**O:9**	**<0.001**	>0.05	>0.05	>0.05
	**CRM_{197}**	**<0.001**	>0.05	>0.05	>0.05
	**saline**	**<0.001**	>0.05	>0.05	>0.05
**bivalent**	**O:4,5-CRM_{197}**	**<0.001**	**<0.05**	>0.05	>0.05
	**O:9-CRM_{197}**	>0.05	>0.05	>0.05	>0.05
	**O:4,5**	**<0.001**	>0.05	>0.05	>0.05
	**O:9**	**<0.001**	>0.05	>0.05	>0.05
	**CRM_{197}**	**<0.001**	>0.05	>0.05	>0.05
	**saline**	**<0.001**	>0.05	>0.05	>0.05
**O:9-CRM_{197}**	**O:4,5-CRM_{197}**	**<0.01**	> 0.05	>0.05	>0.05
	**O:4,5**	**<0.01**	>0.05	>0.05	>0.05
	**O:9**	**<0.001**	>0.05	>0.05	>0.05
	**CRM_{197}**	**<0.01**	>0.05	>0.05	>0.05
	**saline**	**<0.001**	>0.05	>0.05	>0.05
**O:4,5-CRM_{197}**	**O:4,5**	>0.05	>0.05	>0.05	>0.05
	**O:9**	>0.05	>0.05	>0.05	>0.05
	**CRM_{197}**	>0.05	>0.05	>0.05	>0.05
	**saline**	>0.05	>0.05	>0.05	>0.05

[a] O:4,5-CRM_{197}, *S.* Typhimurium O-antigen conjugated with CRM_{197}; O:9-CRM_{197}, *S.* Enteritidis O-antigen conjugated with CRM_{197}; bivalent, O:4,5-CRM_{197} + O:9-CRM_{197}; bivalent + Al^{3+}, O:4,5-CRM_{197} + O:9-CRM_{197} + Al^{3+}; bivalent + Al^{3+} + CpG, O:4,5-CRM_{197} + O:9-CRM_{197} + Al^{3+} + CpG; O:4,5, *S.* Typhimurium O-antigen; O:9, *S.* Enteritidis O-antigen; CRM_{197}: non-toxic mutant of diphtheria toxin.

[b] *P* values assessed using one-way analysis of variance (ANOVA) and Tukey's post test for multiple comparisons.

Table S8. Statistical significance of O:9 – specific serum IgG1, IgG2a, IgG2b, IgG3 titers in CB6F1 mice.

		***P* values [b]**			
**group [a]**	***versus***	**IgG1**	**IgG2a**	**IgG2b**	**IgG3**
**bivalent+Al^{3+}+CpG**	**bivalent+Al^{3+}**	**0.0255**	**<0.0001**	**0.0424**	0.5761

[a] bivalent + Al^{3+}, O:4,5-CRM_{197} + O:9-CRM_{197} + Al^{3+}; bivalent + Al^{3+} + CpG, O:4,5-CRM_{197} + O:9-CRM_{197} + Al^{3+} + CpG

[b] *P* values assessed using two tailed Student t test.

Table S9. Statistical significance of O:4,5 – specific serum IgG titers in C57BL/6 mice.

group [a]	*versus*	*P* values [b]			
		week 2	week 4	week 6	week 7
**bivalent+Al^{3+}+CpG**	**bivalent +Al^{3+}**	>0.05	**<0.05**	>0.05	>0.05
	**O:4,5-CRM$_{197}$ +Al^{3+}**	>0.05	>0.05	**<0.01**	**<0.01**
	**O:4,5-CRM$_{197}$ +Al^{3+}+CpG**	>0.05	>0.05	>0.05	>0.05
	**O:9-CRM$_{197}$ +Al^{3+}**	>0.05	**<0.01**	>0.05	>0.05
	**O:9-CRM$_{197}$ +Al^{3+}+CpG**	>0.05	>0.05	>0.05	>0.05
	**Al^{3+}+CpG**	>0.05	**<0.001**	**<0.001**	**<0.001**
**bivalent+Al^{3+}**	**O:4,5-CRM$_{197}$ +Al^{3+}**	>0.05	>0.05	>0.05	>0.05
	**O:4,5-CRM$_{197}$ +Al^{3+}+CpG**	>0.05	>0.05	>0.05	>0.05
	**O:9-CRM$_{197}$ +Al^{3+}**	>0.05	>0.05	>0.05	>0.05
	**O:9-CRM$_{197}$ +Al^{3+}+CpG**	>0.05	>0.05	>0.05	>0.05
	**Al^{3+}+CpG**	>0.05	**<0.001**	**<0.001**	**<0.001**
**O:9-CRM$_{197}$+Al^{3+}+CpG**	**O:4,5-CRM$_{197}$ +Al^{3+}**	>0.05	>0.05	>0.05	>0.05
	**O:4,5-CRM$_{197}$ +Al^{3+}+CpG**	>0.05	>0.05	>0.05	>0.05
	**O:9-CRM$_{197}$ +Al^{3+}**	>0.05	**<0.05**	>0.05	>0.05
	**Al^{3+}+CpG**	>0.05	**<0.001**	**<0.001**	**<0.001**
**O:9-CRM$_{197}$+Al^{3+}**	**O:4,5-CRM$_{197}$ +Al^{3+}**	>0.05	>0.05	>0.05	>0.05
	**O:4,5-CRM$_{197}$ +Al^{3+}+CpG**	>0.05	>0.05	>0.05	>0.05
	**Al^{3+}+CpG**	>0.05	**<0.001**	**<0.001**	**<0.001**
**O:4,5-CRM$_{197}$+Al^{3+}+CpG**	**O:4,5-CRM$_{197}$ +Al^{3+}**	>0.05	>0.05	>0.05	>0.05
	**Al^{3+}+CpG**	>0.05	**<0.001**	**<0.001**	**<0.001**
**O:4,5-CRM$_{197}$+Al^{3+}**	**Al^{3+}+CpG**	>0.05	**<0.001**	**<0.001**	**<0.001**

[a] O:4,5-CRM$_{197}$ + Al^{3+}, *S.* Typhimurium O-antigen conjugated with CRM$_{197}$ + Al^{3+}; O:4,5-CRM$_{197}$ + Al^{3+} + CpG, *S.* Typhimurium O-antigen conjugated with CRM$_{197}$ + Al^{3+} + CpG; O:9-CRM$_{197}$ + Al^{3+}, *S.* Enteritidis O-antigen conjugated with CRM$_{197}$ + Al^{3+}; O:9-CRM$_{197}$ + Al^{3+} + CpG, *S.* Enteritidis O-antigen conjugated with CRM$_{197}$ + Al^{3+}; bivalent + Al^{3+}, O:4,5-CRM$_{197}$ + O:9-CRM$_{197}$ + Al^{3+}; bivalent + Al^{3+} + CpG, O:4,5-CRM$_{197}$ + O:9-CRM$_{197}$ + Al^{3+} + CpG.

[b] *P* values assessed using one-way analysis of variance (ANOVA) and Tukey's post test for multiple comparisons.

Table S10. Statistical significance of O:4,5 – specific serum IgG titers in C57BL/6 mice.

group [a]	*versus*	***P* values [b]**			
		**week 2**	**week 4**	**week 6**	**week 7**
**bivalent+Al^3+CpG**	**bivalent +Al^{3+}**	0,1948	**0,0066**	**0,0126**	**0,0077**
	**O:4,5-CRM_{197}+Al^{3+}+CpG**	0,3938	0,4993	0,1057	0,0501
	**O:9-CRM_{197} +Al^{3+}+CpG**	0,1703	0,4548	0,1465	**0,0274**
**bivalent+Al^3**	**O:4,5-CRM_{197} +Al^{3+}**	0,4572	0,9869	0,0836	0,1714
	**O:9-CRM_{197}+Al^{3+}**	0,9781	0,5927	0,9409	0,8375
**O:9-CRM_{197}+Al^{3+}+CpG**	**O:9-CRM_{197} +Al^{3+}**	0,8763	**0,0046**	0,2486	0,8539
**O:4,5-CRM_{197}+Al^{3+}+CpG**	**O:4,5-CRM_{197}+Al^{3+}**	0,9536	**0,039**	**0,0247**	0,0601

[a] O:4,5-CRM_{197} + Al^{3+}, *S.* Typhimurium O-antigen conjugated with CRM_{197} + Al^{3+}; O:4,5-CRM_{197} + Al^{3+} + CpG, *S.* Typhimurium O-antigen conjugated with CRM_{197} + Al^{3+} + CpG; O:9-CRM_{197} + Al^{3+}, *S.* Enteritidis O-antigen conjugated with CRM_{197} + Al^{3+}; O:9-CRM_{197} + Al^{3+} + CpG, *S.* Enteritidis O-antigen conjugated with CRM_{197} + Al^{3+}; bivalent + Al^{3+}, O:4,5-CRM_{197} + O:9-CRM_{197} + Al^{3+}; bivalent + Al^{3+} + CpG, O:4,5-CRM_{197} + O:9-CRM_{197} + Al^{3+} + CpG.

[b] *P* values assessed using two tailed Student t test.

Table S11. Statistical significance of O:9 – specific serum IgG titers in C57BL/6 mice.

group [a]	*versus*	*P* values [b] week 2	week 4	week 6	week 7
bivalent+Al^{3+}+CpG	**bivalent +Al^{3+}**	>0.05	>0.05	>0.05	>0.05
	**O:4,5-CRM_{197} +Al^{3+}**	>0.05	**<0.01**	**<0.001**	**<0.001**
	**O:4,5-CRM_{197} +Al^{3+}+CpG**	>0.05	**<0.05**	**<0.001**	**<0.001**
	**O:9-CRM_{197} +Al^{3+}**	>0.05	>0.05	>0.05	>0.05
	**O:9-CRM_{197} +Al^{3+}+CpG**	>0.05	**<0.01**	>0.05	>0.05
	**Al^{3+}+CpG**	>0.05	**<0.01**	**<0.001**	**<0.001**
bivalent+Al^{3+}	**O:4,5-CRM_{197} +Al^{3+}**	>0.05	>0.05	**<0.001**	**<0.001**
	**O:4,5-CRM_{197} +Al^{3+}+CpG**	>0.05	>0.05	**<0.001**	**<0.001**
	**O:9-CRM_{197} +Al^{3+}**	>0.05	>0.05	>0.05	>0.05
	**O:9-CRM_{197} +Al^{3+}+CpG**	>0.05	**<0.001**	>0.05	>0.05
	**Al^{3+}+CpG**	>0.05	>0.05	**<0.001**	**<0.001**
O:9-CRM_{197}+Al^{3+}+CpG	**O:4,5-CRM_{197} +Al^{3+}**	>0.05	**<0.001**	**<0.001**	**<0.001**
	**O:4,5-CRM_{197} +Al^{3+}+CpG**	>0.05	**<0.001**	**<0.001**	**<0.001**
	**O:9-CRM_{197} +Al^{3+}**	>0.05	**< 0.05**	>0.05	>0.05
	**Al^{3+}+CpG**	>0.05	**<0.001**	**< 0.001**	**< 0.001**
O:9-CRM_{197}+Al^{3+}	**O:4,5-CRM_{197} +Al^{3+}**	>0.05	**<0.001**	**<0.001**	**<0.001**
	**O:4,5-CRM_{197} +Al^{3+}+CpG**	>0.05	**<0.01**	**<0.001**	**<0.001**
	**Al^{3+}+CpG**	>0.05	**<0.001**	**<0.001**	**<0.001**
O:4,5-CRM_{197}+Al^{3+}+CpG	**O:4,5-CRM_{197} +Al^{3+}**	>0.05	>0.05	>0.05	>0.05
	**Al^{3+}+CpG**	>0.05	>0.05	>0.05	>0.05
O:4,5-CRM_{197}+Al^{3+}	**Al^{3+}+CpG**	>0.05	>0.05	>0.05	>0.05

[a] O:4,5-CRM_{197} + Al^{3+}, *S.* Typhimurium O-antigen conjugated with CRM_{197} + Al^{3+}; O:4,5-CRM_{197} + Al^{3+} + CpG, *S.* Typhimurium O-antigen conjugated with CRM_{197} + Al^{3+} + CpG; O:9-CRM_{197} + Al^{3+}, *S.* Enteritidis O-antigen conjugated with CRM_{197} + Al^{3+}; O:9-CRM_{197} + Al^{3+} + CpG, *S.* Enteritidis O-antigen conjugated with CRM_{197} + Al^{3+}; bivalent + Al^{3+}, O:4,5-CRM_{197} + O:9-CRM_{197} + Al^{3+}; bivalent + Al^{3+} + CpG, O:4,5-CRM_{197} + O:9-CRM_{197} + Al^{3+} + CpG.

[b] *P* values assessed using one-way analysis of variance (ANOVA) and Tukey's post test for multiple comparisons.

Table S12. Statistical significance of O:9 – specific serum IgG titers in C57BL/6 mice.

group [a]	*versus*	*P* values [b]			
		week 2	week 4	week 6	week 7
**bivalent+Al^3+CpG**	**bivalent +Al^{3+}**	0,4275	0,3177	0,1371	**0,0146**
	**O:4,5-CRM_{197}+Al^{3+}+CpG**	0,2814	**0,0131**	**<0.0001**	**<0.0001**
	**O:9-CRM_{197} +Al^{3+}+CpG**	0,8881	**0,0033**	0,4991	0,6728
**bivalent+Al^3**	**O:4,5-CRM_{197} +Al^{3+}**	0,1699	**0,0148**	**0,0002**	**0,0001**
	**O:9-CRM_{197}+Al^{3+}**	0,8079	0,1675	0,3863	0,0514
**O:9-CRM_{197}+Al^{3+}+CpG**	**O:9-CRM_{197} +Al^{3+}**	0,2782	**0,0045**	0,1944	0,9149
**O:4,5-CRM_{197}+Al^{3+}+CpG**	**O:4,5-CRM_{197}+Al^{3+}**	**0,0068**	0,4733	0,4290	0,2386

[a] O:4,5-CRM_{197} + Al^{3+}, *S.* Typhimurium O-antigen conjugated with CRM_{197} + Al^{3+}; O:4,5-CRM_{197} + Al^{3+} + CpG, *S.* Typhimurium O-antigen conjugated with CRM_{197} + Al^{3+} + CpG; O:9-CRM_{197} + Al^{3+}, *S.* Enteritidis O-antigen conjugated with CRM_{197} + Al^{3+}; O:9-CRM_{197} + Al^{3+} + CpG, *S.* Enteritidis O-antigen conjugated with CRM_{197} + Al^{3+}; bivalent + Al^{3+}, O:4,5-CRM_{197} + O:9-CRM_{197} + Al^{3+}; bivalent + Al^{3+} + CpG, O:4,5-CRM_{197} + O:9-CRM_{197} + Al^{3+} + CpG.

[b] *P* values assessed using two tailed Student t test.

Table S13. Statistical significance of O:4,5 – specific serum IgG1, IgG2b, IgG2c, IgG3 titers in C57BL/6 mice.

		P values [b]			
**group** [a]	***versus***	**IgG1**	**IgG2b**	**IgG2c**	**IgG3**
**bivalent+Al^{3+}+CpG**	**bivalent +Al^{3+}**	>0.05	>0.05	>0.05	>0.05
	**O:4,5-CRM_{197} +Al^{3+}**	**<0.01**	>0.05	>0.05	>0.05
	**O:4,5-CRM_{197} +Al^{3+}+CpG**	>0.05	>0.05	>0.05	>0.05
	**O:9-CRM_{197} +Al^{3+}**	**<0.01**	>0.05	>0.05	>0.05
	**O:9-CRM_{197} +Al^{3+}+CpG**	>0.05	>0.05	>0.05	**<0.05**
	**Al^{3+}+CpG**	**<0.001**	>0.05	>0.05	**<0.01**
**bivalent+Al^{3+}**	**O:4,5-CRM_{197} +Al^{3+}**	>0.05	>0.05	>0.05	>0.05
	**O:4,5-CRM_{197} +Al^{3+}+CpG**	>0.05	**<0.05**	>0.05	>0.05
	**O:9-CRM_{197} +Al^{3+}**	>0.05	>0.05	>0.05	>0.05
	**O:9-CRM_{197} +Al^{3+}+CpG**	>0.05	**<0.05**	**<0.05**	>0.05
	**Al^{3+}+CpG**	**<0.001**	>0.05	>0.05	>0.05
**O:9-CRM_{197}+Al^{3+}+CpG**	**O:4,5-CRM_{197} +Al^{3+}**	>0.05	>0.05	>0.05	>0.05
	**O:4,5-CRM_{197} +Al^{3+}+CpG**	>0.05	>0.05	>0.05	>0.05
	**O:9-CRM_{197} +Al^{3+}**	>0.05	>0.05	>0.05	>0.05
	**Al^{3+}+CpG**	**<0.001**	>0.05	>0.05	>0.05
**O:9-CRM_{197}+Al^{3+}**	**O:4,5-CRM_{197} +Al^{3+}**	>0.05	>0.05	>0.05	>0.05
	**O:4,5-CRM_{197} +Al^{3+}+CpG**	>0.05	>0.05	>0.05	>0.05
	**Al^{3+}+CpG**	**<0.001**	>0.05	>0.05	>0.05
**O:4,5-CRM_{197}+Al^{3+}+CpG**	**O:4,5-CRM_{197} +Al^{3+}**	>0.05	>0.05	>0.05	>0.05
	**Al^{3+}+CpG**	**<0.001**	>0.05	>0.05	>0.05
**O:4,5-CRM_{197}+Al^{3+}**	**Al^{3+}+CpG**	**<0.001**	>0.05	>0.05	>0.05

[a] O:4,5-CRM_{197} + Al^{3+}, *S.* Typhimurium O-antigen conjugated with CRM_{197} + Al^{3+}; O:4,5-CRM_{197} + Al^{3+} + CpG, *S.* Typhimurium O-antigen conjugated with CRM_{197} + Al^{3+} + CpG; O:9-CRM_{197} + Al^{3+}, *S.* Enteritidis O-antigen conjugated with CRM_{197} + Al^{3+}; O:9-CRM_{197} + Al^{3+} + CpG, *S.* Enteritidis O-antigen conjugated with CRM_{197} + Al^{3+}; bivalent + Al^{3+}, O:4,5-CRM_{197} + O:9-CRM_{197} + Al^{3+}; bivalent + Al^{3+} + CpG, O:4,5-CRM_{197} + O:9-CRM_{197} + Al^{3+} + CpG.

[b] *P* values assessed using one-way analysis of variance (ANOVA) and Tukey's post test for multiple comparisons.

Table S14. Statistical significance of O:4,5 – specific serum IgG1, IgG2b, IgG2c, IgG3 titers in C57BL/6 mice.

		P values [b]			
**group** [a]	***versus***	**IgG1**	**IgG2b**	**IgG2c**	**IgG3**
**bivalent+Al^{3}+CpG**	**bivalent +Al^{3+}**	**0.0084**	0.0109	**0.0175**	**0.0008**
	**O:4,5-CRM_{197}+Al^{3+}+CpG**	**0.0178**	0.3019	0.5062	0.1080
	**O:9-CRM_{197} +Al^{3+}+CpG**	**0.0011**	0.1950	0.0980	**0.0002**
**bivalent+Al^{3}**	**O:4,5-CRM_{197} +Al^{3+}**	0.6723	0.2054	0.0830	0.6616
	**O:9-CRM_{197}+Al^{3+}**	0.5849	0.0162	0.0591	0.4533
**O:9-CRM_{197}+Al^{3+}+CpG**	**O:9-CRM_{197} +Al^{3+}**	0.5517	0.0864	**0.0302**	0.3466
**O:4,5-CRM_{197}+Al^{3+}+CpG**	**O:4,5-CRM_{197}+Al^{3+}**	0.3311	0.1017	0.2010	0.7536

[a] O:4,5-CRM_{197} + Al^{3+}, *S.* Typhimurium O-antigen conjugated with CRM_{197} + Al^{3+}; O:4,5-CRM_{197} + Al^{3+} + CpG, *S.* Typhimurium O-antigen conjugated with CRM_{197} + Al^{3+} + CpG; O:9-CRM_{197} + Al^{3+}, *S.* Enteritidis O-antigen conjugated with CRM_{197} + Al^{3+}; O:9-CRM_{197} + Al^{3+} + CpG, *S.* Enteritidis O-antigen conjugated with CRM_{197} + Al^{3+}; bivalent + Al^{3+}, O:4,5-CRM_{197} + O:9-CRM_{197} + Al^{3+}; bivalent + Al^{3+} + CpG, O:4,5-CRM_{197} + O:9-CRM_{197} + Al^{3+} + CpG.

[b] *P* values assessed using two tailed Student t test.

Table S15. Statistical significance of O:9 – specific serum IgG1, IgG2b, IgG2c, IgG3 titers in C57BL/6 mice.

group [a]	*versus*	*P* values [b]			
		IgG1	IgG2b	IgG2c	IgG3
**bivalent+Al^{3+}+CpG**	**bivalent +Al^{3+}**	>0.05	>0.05	>0.05	>0.05
	**O:4,5-CRM_{197} +Al^{3+}**	**<0.001**	>0.05	>0.05	>0.05
	**O:4,5-CRM_{197} +Al^{3+}+CpG**	**<0.001**	>0.05	>0.05	>0.05
	**O:9-CRM_{197} +Al^{3+}**	>0.05	>0.05	>0.05	>0.05
	**O:9-CRM_{197} +Al^{3+}+CpG**	>0.05	>0.05	>0.05	>0.05
	**Al^{3+}+CpG**	**<0.001**	>0.05	>0.05	>0.05
**bivalent+Al^{3+}**	**O:4,5-CRM_{197} +Al^{3+}**	**<0.001**	>0.05	>0.05	>0.05
	**O:4,5-CRM_{197} +Al^{3+}+CpG**	**<0.01**	**<0.05**	>0.05	>0.05
	**O:9-CRM_{197} +Al^{3+}**	>0.05	>0.05	>0.05	>0.05
	**O:9-CRM_{197} +Al^{3+}+CpG**	>0.05	>0.05	>0.05	>0.05
	**Al^{3+}+CpG**	**<0.01**	>0.05	>0.05	>0.05
**O:9-CRM_{197}+Al^{3+}+CpG**	**O:4,5-CRM_{197} +Al^{3+}**	**<0.01**	>0.05	>0.05	>0.05
	**O:4,5-CRM_{197} +Al^{3+}+CpG**	**<0.05**	>0.05	>0.05	>0.05
	**O:9-CRM_{197} +Al^{3+}**	>0.05	>0.05	>0.05	>0.05
	**Al^{3+}+CpG**	**<0.05**	>0.05	>0.05	>0.05
**O:9-CRM_{197}+Al^{3+}**	**O:4,5-CRM_{197} +Al^{3+}**	**<0.001**	>0.05	>0.05	>0.05
	**O:4,5-CRM_{197} +Al^{3+}+CpG**	**<0.001**	**<0.05**	>0.05	>0.05
	**Al^{3+}+CpG**	**<0.001**	>0.05	>0.05	>0.05
**O:4,5-CRM_{197}+Al^{3+}+CpG**	**O:4,5-CRM_{197} +Al^{3+}**	>0.05	>0.05	>0.05	>0.05
	**Al^{3+}+CpG**	>0.05	>0.05	>0.05	>0.05
**O:4,5-CRM_{197}+Al^{3+}**	**Al^{3+}+CpG**	>0.05	>0.05	>0.05	>0.05

[a] O:4,5-CRM_{197} + Al^{3+}, *S.* Typhimurium O-antigen conjugated with CRM_{197} + Al^{3+}; O:4,5-CRM_{197} + Al^{3+} + CpG, *S.* Typhimurium O-antigen conjugated with CRM_{197} + Al^{3+} + CpG; O:9-CRM_{197} + Al^{3+}, *S.* Enteritidis O-antigen conjugated with CRM_{197} + Al^{3+}; O:9-CRM_{197} + Al^{3+} + CpG, *S.* Enteritidis O-antigen conjugated with CRM_{197} + Al^{3+}; bivalent + Al^{3+}, O:4,5-CRM_{197} + O:9-CRM_{197} + Al^{3+}; bivalent + Al^{3+} + CpG, O:4,5-CRM_{197} + O:9-CRM_{197} + Al^{3+} + CpG.

[b] *P* values assessed using one-way analysis of variance (ANOVA) and Tukey's post test for multiple comparisons.

Table S16. Statistical significance of O:9 – specific serum IgG1, IgG2b, IgG2c, IgG3 titers in C57BL/6 mice.

		***P* values [b]**			
**group [a]**	***versus***	**IgG1**	**IgG2b**	**IgG2c**	**IgG3**
**bivalent+Al^{3}+CpG**	**bivalent +Al^{3+}**	0.2192	0.0901	0.1660	0.5899
	**O:4,5-CRM_{197}+Al^{3+}+CpG**	**0.0005**	**0.0074**	0.3865	0.5899
	**O:9-CRM_{197} +Al^{3+}+CpG**	0.5493	0.0901	0.1660	0.5013
**bivalent+Al^{3}**	**O:4,5-CRM_{197} +Al^{3+}**	**<0.0001**	1	0.5899	1
	**O:9-CRM_{197}+Al^{3+}**	0.4023	1	1	0.7424
**O:9-CRM_{197}+Al^{3+}+CpG**	**O:9-CRM_{197} +Al^{3+}**	0.4657	0.1735	0.1658	1
**O:4,5-CRM_{197}+Al^{3+}+CpG**	**O:4,5-CRM_{197}+Al^{3+}**	**0.0273**	**0.0003**	**0.0323**	1

[a] O:4,5-CRM_{197} + Al^{3+}, *S.* Typhimurium O-antigen conjugated with CRM_{197} + Al^{3+}; O:4,5-CRM_{197} + Al^{3+} + CpG, *S.* Typhimurium O-antigen conjugated with CRM_{197} + Al^{3+} + CpG; O:9-CRM_{197} + Al^{3+}, *S.* Enteritidis O-antigen conjugated with CRM_{197} + Al^{3+}; O:9-CRM_{197} + Al^{3+} + CpG, *S.* Enteritidis O-antigen conjugated with CRM_{197} + Al^{3+}; bivalent + Al^{3+}, O:4,5-CRM_{197} + O:9-CRM_{197} + Al^{3+}; bivalent + Al^{3+} + CpG, O:4,5-CRM_{197} + O:9-CRM_{197} + Al^{3+} + CpG.

[b] *P* values assessed using two tailed Student t test.
